# Dimension constraints improve hypothesis testing for large-scale, graph-associated, brain-image data

**DOI:** 10.1093/biostatistics/kxab001

**Published:** 2021-02-22

**Authors:** Tien Vo, Akshay Mishra, Vamsi Ithapu, Vikas Singh, Michael A Newton

**Affiliations:** Department of Biostatistics and Medical Informatics, University of Wisconsin at Madison 610 Walnut Street, Madison, WI, USA; Department of Biostatistics and Medical Informatics, University of Wisconsin at Madison 610 Walnut Street, Madison, WI, USA; Department of Biostatistics and Medical Informatics, University of Wisconsin at Madison 610 Walnut Street, Madison, WI, USA; Department of Biostatistics and Medical Informatics, University of Wisconsin at Madison 610 Walnut Street, Madison, WI, USA; Department of Biostatistics and Medical Informatics, University of Wisconsin at Madison 610 Walnut Street, Madison, WI, USA

**Keywords:** Empirical Bayes, Graph-respecting partition, GraphMM, Image analysis, Local false-discovery rate, Mixture model

## Abstract

For large-scale testing with graph-associated data, we present an empirical Bayes mixture technique to score local false-discovery rates (FDRs). Compared to procedures that ignore the graph, the proposed Graph-based Mixture Model (GraphMM) method gains power in settings where non-null cases form connected subgraphs, and it does so by regularizing parameter contrasts between testing units. Simulations show that GraphMM controls the FDR in a variety of settings, though it may lose control with excessive regularization. On magnetic resonance imaging data from a study of brain changes associated with the onset of Alzheimer’s disease, GraphMM produces greater yield than conventional large-scale testing procedures.

## 1. Introduction

Empirical Bayesian methods provide a useful approach to large-scale hypothesis testing in genomics, brain imaging, and other application areas. Often, these methods are applied relatively late in the data-analysis pipeline, after p-values, test statistics, or other summary statistics are computed for each testing unit. Essentially, the analyst performs univariate testing *en masse*. The final unit-specific scores and discoveries depend upon the chosen empirical Bayesian method, which accounts for the collective properties of the separate statistics to gain an advantage (e.g., Storey, 2003; Efron, 2007; Stephens, 2017). These methods are effective but may be underpowered in some applied problems when the underlying effects are relatively weak. Motivated by tasks in neuroscience, we describe an empirical Bayesian approach that operates earlier in the data-analysis pipeline and that leverages regularities achieved by constraining the dimension of the parameter space. Our approach is restricted to data sets in which the variables constitute nodes of a known, undirected graph, which we use to guide regularization. We report simulation and empirical studies with structural magnetic resonance imaging to demonstrate encouraging operating characteristics of the new methodology. We conjecture that power is gained for graph-associated data by moving upstream in the data reduction process and by recognizing low complexity parameter states.

**Fig. 1:**
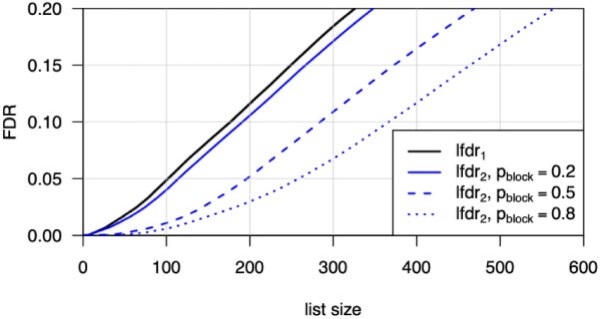
False-discovery rate of list (vertical) as a function of list size (horizontal) for various testing procedures. ${\rm lfdr}_1$ refers to the procedure to list the unit if the local FDR $P(\mu_{X_1}=\mu_{Y_1}|X_1,Y_1)$ is sufficiently small (black). Blue lines refer to the operating characteristics when using ${\rm lfdr}_2$ which is $P(\mu_{X_1}=\mu_{Y_1}| X_1, X_2, Y_1, Y_2)$, for various probabilities $p_{\rm block}$ that the two units share parameters. By accounting for blocking, we may report more discoveries at a given FDR. The synthetic system has 10$^4$ unit pairs and marginal $p_0=P( \mu_{X_1}=\mu_{Y_1} ) = 0.8$; as the list size increases all curves approach $p_0$.

The following toy problem illustrates in a highly simplified setting the phenomenon we leverage for improved power. Suppose we have two sampling conditions, and two variables measured in each condition, say $X_1$ and $X_2$ in the first condition and $Y_1$ and $Y_2$ in the second. We aim to test the null hypothesis that $X_1$ and $Y_1$ have the same expected value; say $H_0: \mu_{X_1} = \mu_{Y_1}$. Conditional upon target values $\mu_{X_1}$, $\mu_{Y_1}$ and nuisance mean values $\mu_{X_2}$ and $\mu_{Y_2}$, the four observations are mutually independent, with normal distributions and some constant, known variance $\sigma^2$. We further imagine that these four variables are part of a larger system, throughout which the distinct expected values themselves fluctuate, say according to a standard normal distribution. Within this structure, a test of $H_0$ may be based upon the local false-discovery rate (FDR)

$$
\begin{eqnarray*}
{\rm lfdr}_1 =
P(H_0|X_1,Y_1) = \frac{p_0 f(X_1,Y_1)}{p_0 f(X_1, Y_1) + (1-p_0) g(X_1) g(Y_1)},
\end{eqnarray*}
$$

where we are mixing discretely over null (with probability $p_0$) and non-null cases. Here the across-system variation in expected values may be handled analytically and integrated out; thus in this predictive distribution $g(x)=\int N(x|\mu,\sigma^2) \, N(\mu|0,1) \,{\rm d}\mu$ is the density of a mean 0 normal distribution with variance $1+\sigma^2$; and $f(x,y)=\int N(x|\mu,\sigma^2) \, N(y|\mu, \sigma^2) \, N(\mu|0,1) \, {d}\mu$ is the bivariate normal density with margins $g$ and with correlation $1/(1+\sigma^2)$ between $X_1$ and $Y_1$ (in the integrals, $N$ is the normal density). In considering data $X_2$ and $Y_2$ on the second variable, it may be useful to suppose that the expected values here are no different from their counterparts on the first variable. We say the variables are blocked if both $\mu_{X_1} = \mu_{X_2}$ and $\mu_{Y_1} = \mu_{Y_2}$, and we consider this a discrete possibility that occurs with probability $p_{\rm block}$ throughout the system, independently of $H_0$. In the absence of blocking, there is no information in $X_2$ and $Y_2$ that could inform the test of $H_0$ (considering the independence assumptions). In the presence of blocking, however, data on these second variables are highly relevant. Treating blocking as random across the system, we would score $H_0$ using the local FDR ${\rm lfdr}_2 = P( H_0 | X_1, X_2, Y_1, Y_2 )$, which requires for evaluation the consideration of a 4-variate normal and joint discrete mixing over the blocking and null states. Figure 1 shows the result of simulating a system with $10^4$ variable pairs, where the marginal null frequency $p_0=0.8$, $\sigma^2 = 1/2$, and the blocking rate $p_{\rm block}$ varies over three possibilities. Shown is the FDR of the list (i.e., the mean of local FDRs for units on the list) formed by ranking instances by either ${\rm lfdr}_1$ or ${\rm lfdr}_2$. The finding in this toy problem is that power for detecting differences between $\mu_{X_1}$ and $\mu_{Y_1}$ increases by accounting for the blocking, since the list of discovered non-null cases by ${\rm lfdr}_2$ is larger for a given FDR than the list constructed using ${\rm lfdr}_1$. In other words, when the dimension of the parameter space is constrained, more data become relevant to the test of $H_0$ and power increases.

Our interest in large-scale testing arises from work with structural magnetic resonance imaging (MRI) data measured in studies of brain structure and function, as part of the Alzheimer’s Disease Neuroimaging Initiative (ADNI-2) (Weiner and Veitch, 2015). MRI provides a detailed view of brain atrophy and has become an integral to the clinical assessment of patients suspected to have Alzheimer’s disease (AD) (e.g., Vemuri and Jack, 2010; Moller *and others*, 2013). In studies to understand disease onset, a central task has been to identify brain regions that exhibit statistically significant differences between various clinical groups, while accounting for technical and biological sources of variation affecting MRI scans. Existing work in large-scale testing for neuroimaging has considered thresholds on voxel-wise test statistics to control a specified false positive rate and maintain testing power (Nichols, 2012). Two widely used approaches are family-wise error control using random field theory (e.g., Worsley *and others*, 2004) and FDR control using Benjamin–Hochberg procedure (Benjamini and Hochberg, 1995; Genovese *and others*, 2002). The former is based on additional assumptions about the spatial smoothness of the MRI signal, which may not be supported empirically (Eklund *and others*, 2016). Both parametric and nonparametric voxel-wise tests are available in convenient neuroimaging software systems (Penny *and others*, 2007; Nichols). Recently, Tansey *and others* (2018) presented an FDR tool that processes unit-specific test statistics in a way to spatially smooth the estimated prior proportions. As the clinical questions of interest move towards identifying early signs of AD, the changes in average brain profiles between conditions invariably become more subtle and increasingly hard to detect; the result is that very few voxels or brain regions may be detected as significantly different by standard methods.

Making a practical tool from the blocking phenomenon (Figure 1) requires that a number of modeling and computational issues be resolved. Others have recognized the potential and have designed computationally intensive Bayesian approaches based on Markov chain Monte Carlo (Do *and others*, 2005; Dahl and Newton, 2007; Dahl *and others*, 2008; Kim *and others*, 2009). We seek simpler methodology and develop a specific case in which data are organized by a known undirected graph; then blocking may occur between one testing unit and another unit nearby in the graph. For flexibility, we avoid the often-used product-partition assumption, and we rely on graph-localization to reduce computational complexity: after setting global hyperparameters, a unit’s local FDR is computed from data on that unit as well on units in a local subgraph. The resulting tool we call GraphMM, for graph-based mixture model. It is deployed as an R package available at https://github.com/tienv/GraphMM/. We investigate its properties using a variety of synthetic-data scenarios, and we apply it to identify statistically significant changes in brain structure associated with the onset of mild cognitive impairment. Details not found in the following sections are included in Supplementary material available at *Biostatistics* online.

## 2. Methods

### 2.1. Data structure and inference problem

Let $G=(V,E)$ denote a simple, connected, undirected graph with vertex set $V = \{1, 2, ..., N\}$ and edge set $E$, and consider partitions of $V$, such as $\Psi = \{ b_1, ..., b_K\}$; that is, blocks (also called clusters) $b_k$ constitute non-empty disjoint subsets of $V$ for which $\cup_{k=1}^K b_k = V$. In the application in Section 3.2, vertices correspond to voxels at which brain-image data are measured, edges connect spatially neighboring voxels, and the partition conveys a dimension-reducing constraint. The framework is quite general and includes, for example, interesting problems from genomics and molecular biology. Recall that for any subset $b \subset V$, the induced subgraph $G_b = (b, E_b)$, where $E_b$ contains all edges $e=(v_1,v_2)$ for which $e \in E$ and $v_1, v_2 \in b$. For use in constraining a parameter space, we introduce the following property:

Property 2.1 (Graph-respecting partition) A partition $\Psi$ respects $G$, or $\Psi$ is graph respecting, if for all $b_k \in \Psi$, the induced graph $G_{b_k}$ is connected.

**Fig. 2:**
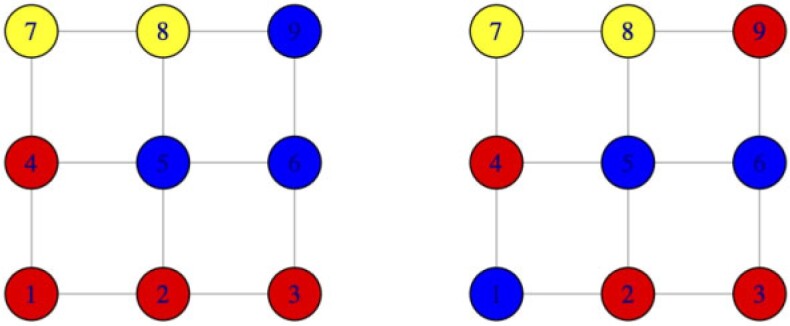
Examples of partitions on a graph. Different colors represent different blocks. The partition on the left is graph respecting while the one on the right is not (e.g., the blue nodes induces a subgraph with two components). There are 1434 such graph-respecting partitions of this $3 \times 3$ lattice. They have a median number of four blocks.

Figure 2 presents a simple illustration; a spanning-tree representation turns out to be useful in computations (Supplementary material available at *Biostatistics* online). It becomes relevant to statistical modeling that the size of the set of graph-respecting partitions, though large, still is substantially smaller than the set of all partitions as the graph itself becomes less complex. For example there are 21 147 partitions of nine objects (the 9th Bell number), but if these objects are arranged as vertices of a regular $3 \times 3$ lattice graph, then there are only 1434 graph-respecting partitions.

In our setting, the graph $G$ serves as a known object that provides structure to a data set being analyzed for the purpose of a two-group comparison. This is in contrast, for example, to graphical-modeling settings where the possibly unknown graph holds the dependency patterns of the joint distribution. We write the two-group data as $\boldsymbol{X} = (X_{v,m})$ and $\boldsymbol{Y} = (Y_{v,r})$, where $v \in V$, $ m=1,\dots, M_X $ and $ r=1,\dots, M_Y$. Here, $M_X$ and $M_Y$ denote the numbers of replicate samples in both groups. In Section 3.2, for example, $m$ indexes the brain of a normal control subject and $r$ indexes the brain of a subject with mild cognitive impairment. For convenience, let $\boldsymbol{X_{m}}=( X_{v,m},\; v \in V )$ and $\boldsymbol{Y_{r}}=(Y_{v,r},\; v \in V )$ denote the across-graph samples on subjects $m$ and $r$, which we treat as identically distributed within group and mutually independent over $m$ and $r$ owing to the two-group, unpaired experimental design.

Our methodology tests for changes between the two groups in the expected-value vectors: $\boldsymbol{\mu_X} = E( \boldsymbol{X_{m}} ) = (\mu_{X_1}, \dots, \mu_{X_N}) $ and $\boldsymbol{\mu_Y} = E( \boldsymbol{Y_{r}} ) = (\mu_{Y_1}, \dots, \mu_{Y_N})$. Specifically, we aim to test, for any vertex $v \in V$, $H_{0, v}: \mu_{X_v} = \mu_{Y_v}$ versus $H_{1, v}: \mu_{X_v} \neq \mu_{Y_v}$. We seek to gain statistical power over contemporary testing procedures by imposing a dimension constraint on the expected values. Although it is not required to be known or even estimated, we suppose there exists a graph-respecting partition $\Psi=\{b_k\}$ that constrains the expected values:

(2.1) \begin{align*} \label{meanclusX} &\begin{cases} \mu_{X_v} = \mu_{X_u} \quad \quad \textrm{if for some $k$, both}\, v, u \in b_k \\ \mu_{X_v} \neq \mu_{X_u} \quad \quad \textrm{if} \, v, u \textrm{ belong to different blocks } \end{cases}\\ & \begin{cases} \mu_{Y_v} = \mu_{Y_u} \quad \quad \textrm{if for some $k$, both}\, v, u \in b_k \\ \mu_{Y_v} \neq \mu_{Y_u} \quad \quad \textrm{if} \, v, u \textrm{ belong to different blocks } \end{cases}.\nonumber \end{align*}

All vertices $v$ in block $b_k$ have a common mean in the first group, say $\varphi_k$, and a common mean $\nu_k$ in the second group. The contrast on test, then, is $\delta_k = \nu_k - \varphi_k$; together with $\Psi$, the binary vector $\boldsymbol{\Delta} = (\Delta_1, \dots, \Delta_K )$ holding indicators $\Delta_k = 1[ \delta_k \neq 0 ]$ is equivalent to knowing whether or not $H_{0,v}$ is true for each vertex $v$. When data are consistent with a partition $\Psi$ in which the number of blocks $K$ is small compared to the number of vertices $N$, then it may be possible to leverage this reduced parameter-space complexity for the benefit of hypothesis-testing power.

### 2.2. Graph-based mixture model

#### 2.2.1. Discrete mixing

We adopt an empirical Bayes, mixture-based testing approach, which requires that for each vertex we compute a local FDR:

(2.2) \begin{equation*} \label{postprob} l_v := P(H_{0, v} | \boldsymbol{X}, \boldsymbol{Y}) = \sum_{\Psi, \boldsymbol{\Delta}} (1-\Delta_k) {\rm 1}\kern-0.24em{\rm I}( v \in b_k)P(\boldsymbol{\Delta}, \Psi|\boldsymbol{X}, \boldsymbol{Y}). \end{equation*}

Our list $\mathcal L$ of discovered (non-null) vertices is $\mathcal{L} = \{ v: l_v \leq c \}$ for some threshold $c$. Conditional on the data, the expected rate of type-I errors within $\mathcal L$ is dominated by the threshold $c$ (Efron, 2007; Newton *and others*, 2004). The sum in (2.2) is over the finite set of pairs of partitions $\Psi$ and block-change indicator vectors $\boldsymbol{\Delta}$. This set is intractably large for even moderate-sized graphs: we present here computations in the context of very small graphs. For each vertex $v$ in the original graph, we consider a small local subgraph in which $v$ is the central vertex, and we deploy GraphMM on this local subgraph. This simplification suppresses any direct effect that non-local data may have on inference at vertex $v$, but the allowance for the influence of local data is of course greater than that imparted by standard large-scale methods.

Summing in (2.2) delivers marginal posterior inference, and thus a mechanism for borrowing strength among vertices $v$. By Bayes’s rule, $P(\boldsymbol{\Delta}, \Psi|\boldsymbol{X}, \boldsymbol{Y}) \propto f(\boldsymbol{X}, \boldsymbol{Y} | \boldsymbol{\Delta}, \Psi ) \, P(\boldsymbol{\Delta}, \Psi )$, and both the mass $P(\boldsymbol{\Delta}, \Psi )$ and the predictive density $f(\boldsymbol{X}, \boldsymbol{Y} | \boldsymbol{\Delta}, \Psi )$ need to be specified to compute inference summaries. Various modeling approaches present themselves. For example, we could reduce data per vertex to a test statistic (e.g., t-statistic) and model the predictive density nonparametrically, as in locfdr (Efron, 2007). We could reduce data per vertex less severely, retaining effect estimates and estimated standard errors, as in adaptive shrinkage (Stephens, 2017). By contrast, we adopt an explicit parametric-model formulation for the predictive distribution of data given the discrete state $(\Psi, \boldsymbol{\Delta})$. It restricts the sampling model to be Gaussian but allows general covariance among vertices and is not reliant on the product-partition assumption commonly used in partition-based models (Barry and Hartigan, 1992). For $P(\Psi,\Delta)$, we specify $P(\Psi) \propto 1$, we encode independent and identically distributed block-specific Bernoulli$(p_0)$ indicators in $P(\Delta|\Psi)$, and we use univariate empirical-Bayes techniques to estimate $p_0$.

#### 2.2.2. Predictive density given discrete structure

We take a multivariate Gaussian sampling model:

$$
\begin{eqnarray*}
\boldsymbol{X_{m}} | \boldsymbol{\mu_X}, U, \Psi, \boldsymbol{\Delta} \sim_{\rm i.i.d.} \mathcal{N}(\boldsymbol{\mu_X}, U) \quad m = 1, \dots, M_X, \quad
\boldsymbol{Y_r} | \boldsymbol{\mu_Y}, W, \Psi, \boldsymbol{\Delta} \sim_{\rm i.i.d.} \mathcal{N}(\boldsymbol{\mu_Y}, W) \quad r = 1, \dots, M_Y.
\end{eqnarray*}
$$

We we place a conjugate inverse Wishart prior distribution on covariance matrices: $ U | \Psi, \boldsymbol{\Delta}, \boldsymbol{\mu_X}, \boldsymbol{\mu_Y} \sim \mathcal{IW}(A, \textrm{df})$, and $W | \Psi, \boldsymbol{\Delta}, \boldsymbol{\mu_X}, \boldsymbol{\mu_Y} \sim \mathcal{IW}(B, \textrm{df})$. In general, there is no simple conjugate reduction for predictive densities owing to the less-than-full dimension of free parameters in $\boldsymbol{\mu_X}$ and $\boldsymbol{\mu_Y}$. On these free parameters, we further specify independent Gaussian priors: $\varphi_k \sim \mathcal{N}\left(\mu_0, \tau^2 \right)$ and, for $\Delta_k \neq 0$, $\delta_k \sim \mathcal{N}\left(\delta_0, \sigma^2\right)$. Hyperparameters in GraphMM include scalars $\delta_0$, $\mu_0$, $\tau^2$, $\sigma^2$, df, and matrices $A$, $B$, which we estimate from data across the whole graph following the empirical-Bayes approach (see Supplementary material available at *Biostatistics* online, Section 2.2).

The above specification induces a joint density $f( \boldsymbol{X}, \boldsymbol{Y}, \boldsymbol{\mu_X}, \boldsymbol{\mu_Y}, U, W | \boldsymbol{\Delta}, \Psi)$. For the purpose of hypothesis testing, we need to marginalize most variables, since $H_{0,v}$ is equivalent to $\Delta_k = 0$ and $v \in b_k$ for block $b_k$ in partition $\Psi$, and local FDRs require marginal posterior probabilities. Integrating out inverse Wishart distributions over the covariance matrices is possible analytically. We find:

(2.3) \begin{eqnarray*} \label{lkh} f(\boldsymbol{X}, \boldsymbol{Y} \,\, |\,\, \boldsymbol{\mu_X}, \boldsymbol{\mu_Y}, \boldsymbol{\Delta}, \Psi) & = & C \frac{|A|^{\frac{\text{df}}{2}} |B|^{\frac{\text{df}}{2}}}{|\widetilde{A}|^{\frac{\text{df}+M_X}{2}} |\widetilde{B}|^{\frac{\text{df}+M_Y}{2}}} \end{eqnarray*}

where

$$\begin{array}{c} S_{1}=\frac{1}{M_{X}-1}\sum_{m=1}^{M_{X}}(X_{m}-\bar{X})(X_{m}-\bar{X}{)}^{T},\;S_{2}=(\bar{X}-\mu_{X})(\bar{X}-\mu_{X}{)}^{T}\\ T_{1}=\frac{1}{M_{Y}-1}\sum_{r=1}^{M_{Y}}(Y_{r}-\bar{Y})(Y_{r}-\bar{Y}{)}^{T},\;T_{2}=(\bar{Y}-\mu_{Y})(\bar{Y}-\mu_{Y}{)}^{T}\\ \tilde{A}=A+(M_{X}-1)S_{1}+M_{X}S_{2},\;\tilde{B}=B+(M_{Y}-1)T_{1}+M_{Y}T_{2}, \end{array}$$

and where $C$ is a normalizing constant. In the above, $|.|$ denotes matrix determinant, $ \overline{\boldsymbol{X}} = \frac{1}{M_X}\sum_{m=1}^{M_X} \boldsymbol{X_m}$, $\overline{\boldsymbol{Y}} = \frac{1}{M_Y} \sum_{r=1}^{M_Y} \boldsymbol{Y_r},$ and $S_1$ and $T_1$ are sample covariance matrices of $\boldsymbol{X}$ and $\boldsymbol{Y}$. In (2.3), there is conditional independence of data from the two conditions given the means but marginal to the unspecified covariance matrices. When a specific adjustment of each sample covariance is of full rank, we can use the Laplace approximation to numerically integrate the freely varying means in order to obtain the marginal predictive density $f(\boldsymbol{X}, \boldsymbol{Y} | \boldsymbol{\Delta}, \Psi)$ (Supplementary material available at *Biostatistics* online, Equation 2.3). Computations would simplify under a product-partition assumption, but we found in preliminary numerical experiments that various data sets are not consistent with this simplified dependence pattern, and we deploy the general form above. The marginal predictive density depends on the two-sample data through fixed-dimensional sufficient statistics; we expect some robustness of the methodology to non-normality of the data owing to central-limit effects on these sufficient statistics in case of moderate to large sample sizes (Figures S11 and S12 of the Supplementary material available at *Biostatistics* online).

## 3. Results

### 3.1. Brain MRI study: ADNI-2

Our primary evaluation of GraphMM is through a set of calculations designed around a motivating data set from the Alzheimer’s Disease Neuroimaging Initiative 2 (ADNI-2). Briefly, we consider 3D structural brain-imaging data from a group of $M_X=123$ cognitively normal control subjects (CN) and a second group of $M_Y = 148$ subjects suffering from late-stage mild cognitive impairment (MCI), a precursor to Alzheimer’s disease (AD). Gray matter tissue probability maps derived from the co-registered T1-weighted magnetic resonance imaging (MRI) data were pre-processed using the voxel-based morphometry (VBM) toolbox in Statistical Parametric Mapping software (SPM, http://www.fil.ion.ucl.ac.uk/spm). Prior to registration to a common template, standard artifact removal and other corrections were performed, as described in Ithapu *and others* (2015). We filtered voxels having very low marginal standard deviation (Bourgon *and others*, 2010), leaving $M_X+M_Y=271$ measurements at each of 464 441 voxels, which reside within a $121\times 121 \times 119$ 3D lattice. We also converted the data to rank-based normal scores prior to comparisons between CN and MCI groups.

### 3.2. Data-driven simulations

To check basic operating characteristics of GraphMM, we construct synthetic data mimicking the size and variation characteristics of a single coronal slice containing $N=5236$ voxels from ADNI-2. In a series of empirically guided generative scenarios, we consider various levels of clustering within the latent expected values and various shifts between synthetic CN and MCI groups. Briefly, we derive blocked latent mean states through spatial clustering, and we use empirical covariances and empirical group shifts to guide these simulations. Supplementary material available at *Biostatistics* online, Section 4, reports further details. We deploy GraphMM using the local $3\times 3$ lattice subgraph centered on each voxel on test; three data sets are generated in each scenario, and error/detection rates are averaged.

When applying GraphMM to each synthetic data set, we estimate hyperparameters from the entire slice and consider discoveries as ${\mathcal L}(c) = \{ v: l_v \leq c \}$ for various thresholds $c$. We call the controlled FDR the mean $\sum_v l_v 1[ v \in {\mathcal L}(c) ]/\sum_v 1[v \in {\mathcal L}(c)]$, as this is the conditional expected rate of type-1 errors on the list, given data (and computable from data). We know the null status in each synthetic case, and so we also call the empirical FDR to be that rate counting latent null indicators; likewise the true positive rate counts the non-null indicators. We compare GraphMM to several contemporary testing methods, including Benjamini–Hochberg correction (BH adj), locfdr, and qvalue (Storey, 2003), which process voxel-specific t-tests. We also compare results to adaptive shrinkage, both the local FDR statistic (ash_lfdr) and the $q$-value (ash_qval). None of these comparators aim to leverage the graphical nature of the data.

**Fig. 3:**
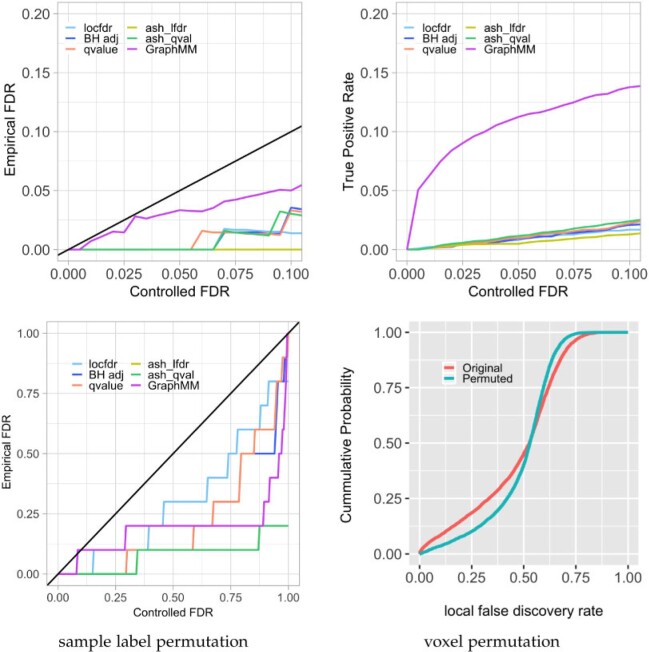
Operating characteristics (top panels) of GraphMM and comparator methods in the first data-driven simulation (12–14 voxels per underlying block), and behavior of GraphMM in two permutation experiments (bottom panels). Dominance by the diagonal line (top left panel) shows that all methods control the FDR in this scenario; the top right panel reveals high sensitivity of GraphMM in this case with relatively large blocks. The sample-label-permutation experiment (lower left) confirms that GraphMM controls FDR in this no-signal case. The voxel-permutation experiment (lower right) confirms that the detection rate is reduced (fewer small local FDRs) when we disrupt the spatial signal.

The first three scenarios vary the underlying size-distribution of blocks, but follow the GraphMM model in the sense that the underlying signal has graph-respecting partitions, and other conditions such as block-level shifts between conditions and multivariate Gaussian errors are satisfied. The top panels of Figure 3 show for the first simulation scenario that all methods on test control the FDR and have sensitivity for relevant signals, though GraphMM has increased power. Figure S3 of the Supplementary material available at *Biostatistics* online shows qualitatively similar results for all three scenarios. The high sensitivity in Scenario 2 may reflect that the prior distribution of block sizes used in the local GraphMM more closely matches the generative situation. Notably, even when this block-size distribution is not aligned with the GraphMM prior, we do not see an inflation of the FDR. Scenarios 4 and 5 are similar to the first cases, however they explore different forms of signals between the two groups; both have an average block size of 4 voxels, but in one case the changed block effects are fewer, relatively strong and in the other case they are more frequent, and relatively weaker (Table S3 of the Supplementary material available at *Biostatistics* online). In both regimes, GraphMM retains its control of FDR and exhibits good sensitivity compared to other methods (Figure S4 of the Supplementary material available at *Biostatistics* online).

GraphMM is designed for the case where partition blocks are graph respecting and the changes between conditions affect entire blocks. Our next numerical experiment checks the robustness of GraphMM when this partition/change structure is violated. Briefly, we had a generative situation similar to the five scenarios above, except that the latent means were not graph respecting on the 2D lattice (details in Supplementary material available at *Biostatistics* online, Section 3.2). Figure S5 of the Supplementary material available at *Biostatistics* online shows that GraphMM continues to control FDR. The modest sensitivity advantage in this case may stem from the fact that the latent means are clustered and low-dimensional, even though blocks may not be fully contiguous.

To further assess the properties of GraphMM, we performed two permutation experiments leveraging the ADNI-2 data. In the first, we permuted the sample labels of the 148 control subjects and 123 late MCI subjects, repeating for ten permuted sets. On each permuted set, we applied various methods to detect differences. All discoveries are false discoveries in this null case. The lower left panel of Figure 3 shows that GraphMM and other methods are correctly recognizing the apparent signals as being consistent with the null hypothesis. The second permutation experiment retains the sample-grouping information, but permutes the voxels within the brain slice on test. This permutation disrupts both spatial measurement dependencies and any spatial structure in the signal. Since GraphMM is leveraging spatially coherent patterns in signal, we expect it to produce fewer statistically significant findings in this voxel-permutation case. The lower right panel of Figure 3 shows this dampening of signal as we expect, when looking at the empirical distribution of statistics $l_v=P(H_{0,v}|X,Y)$.

### 3.3. ADNI-2 data analysis

We seek to identify brain locations (i.e., voxels) that exhibit significant differences in MRI-based gray matter intensity between two disease stages (cognitively normal controls and late MCI), and also assess the extent to which our findings are corroborated by known results on aging and Alzheimer’s disease. First, we applied GraphMM with a $3 \times 3$ local lattice within each of the 119 2D image slices in the coronal direction. For comparison, we applied Statistical non-parametric Mapping toolbox using Matlab, SnPM, which is a popular image analysis method used in neuroscience, and $q$-value with adaptive shrinkage, ashr, which represents an advanced voxel-specific empirical-Bayes method.

**Fig. 4:**
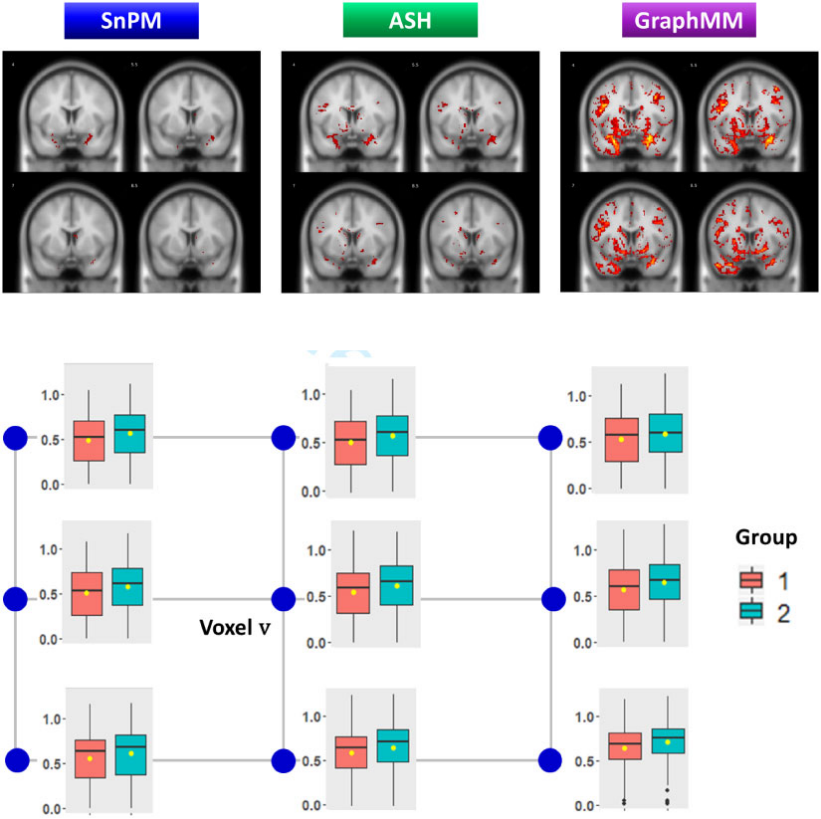
Top panel shows significantly different voxels at 5% FDR (reds to yellows, see text) for four coronal slices, found by statistical non-parametric mapping (SnPM), adaptive shrinkage (ASH) and the proposed GraphMM. Bottom panel shows boxplots for one voxel, $v$, at coordinates $(x = 31, y = 53, z = 23)$ and its neighbors. Voxel $v$ is altered according to GraphMM (lfdr 0.001) but not according to other methods (e.g., ASH $q$-value is 0.138). Similar shifts nearby $v$ lead to the stronger evidence reported by GraphMM.

Figure 4 (top) shows a representative example output for a montage of four coronal slices extracted from the 3D image volume. The color bar (red to yellow), for each method presented, is a surrogate for the strength of some score describing the group-level difference: for instance, for SnPM, the color is scaled based on adjusted $p$-values, for the $q$-value method, it is scaled based on $q$-values, whereas for GraphMM, the color is scaled based on local FDRs $l_v$. While the regions reported as significantly different between controls and late MCI have some overlap between the different methods, GraphMM is able to identify many more significantly different voxels compared to baseline methods, at various FDR thresholds (Figure S6 of the Supplementary material available at *Biostatistics* online). A closer inspection of one case is informative (Figure 4, bottom). Voxel $v$ at coordinates $(x = 31, y = 53, z = 23)$ is not found to be different between control and late MCI according to SnPM (adjusted $p$-value = 0.578) or the ASH $q$-value method ($q$-value = 0.138), but GraphMM reports local FDR $0.001$. The consistent but modest shift in means between control and late MCI in this small spatial region explains the GraphMM finding.

A statistical measure often reported in the neuroimaging literature is the size of spatially connected sets of significantly altered voxels in the 3D lattice (so-called significant clusters). The rationale is that stray (salt and pepper) voxels reported as significantly different may be more likely to be an artifact compared to a group of anatomically clustered voxels. GraphMM performs favorably relative to the baseline methods in that it consistently reports larger significant clusters (Figure S7 of the Supplementary material available at *Biostatistics* online).

To provide some neuroscientific interpretation of the statistical findings, we use the Matlab package *xjview* to link anatomical information associated with significantly altered voxels (Tzourio-Mazoyer *and others*, 2002). Results for the top 15 brain regions are summarized in Table S4 of the Supplementary material available at *Biostatistics* online. GraphMM discovers all the brain regions found by SnPM, with many more significant voxels in each region. The only exception is the hippocampus, where both methods identify a large number of voxels but GraphMM finds fewer significant voxels than SnPM. In addition, there are regions revealed to be significant by GraphMM but not by SnPM, including the precentral gyrus, middle frontal gyrus, inferior frontal gyrus opercular, insular, anterior cingulate, and supramarginal gyrus, which are relevant in the aging and AD literature. GraphMM consolidates known alterations between cognitively normal and late-stage MCI and reveals potentially important new findings.

Reported above are whole-brain results from slice-level runs of GraphMM. We also ran GraphMM over the whole brain for two further choices of local graph: both are simple star graphs with center node equal to the target node on test, and with edges to all first-order neighbors. We entertain the 3D neighborhood, where a typical node has six neighbors, and also a 2D neighborhood having four neighbors (and thus a further subgraph of the $3 \times 3$ lattice subgraph used above. We used a single ASH-estimated value for the overall null frequency $p_0$. Looking at the collection of local FDRs, we see a modest shift in the distribution of local FDR values when they access the 3D information compared to having only the 2D slice information (Figure S9 of the Supplementary material available at *Biostatistics* online); there is also considerable agreement in voxel ranking by the methods (Figure S8 of the Supplementary material available at *Biostatistics* online).

## 4. Discussion

Mass univariate testing is the dominant approach to detect statistically significant changes in comparative brain-imaging studies (e.g., Groppe *and others*, 2011). In such, a classical testing procedure, like the test of a contrast in a regression model, is applied in parallel over all testing units (voxels), leading to a large number of univariate test statistics and p-values. Subsequently, significant voxels are identified through some filter, such as the BH procedure, to control the FDR. The approach can be very effective and has supported numerous important applied studies of brain function. In structural magnetic resonance image studies of Alzheimer’s disease progression, such mass univariate testing has failed in some cases to reveal subtle structural changes between phenotypically distinct patient populations. An underlying problem is limited statistical power for relatively small effects, even with possibly hundreds of subjects per group. Power may be recovered by empirical Bayes procedures that leverage various properties of the collection of tests. The proposed GraphMM method recognizes simplified parameter states when they exist among graphically related testing units. We deploy GraphMM locally in the system-defining graph by separately processing a small subgraph for each testing unit, while allowing hyper-parameters to be estimated globally from all testing units. Essentially, we provide an explicit and flexible joint probability model for all data on each subgraph (Equation 2.3). The model entails a discrete parameter state on this subgraph, which describes how the nodes on the subgraph are partitioned into blocks, and whether or not each block is shifted between the two sampling conditions being compared. By deriving local FDR computations on a relatively small subgraph for each testing unit, we simplify computations and we share perhaps the most relevant information that is external to that testing unit. Numerical experiments confirm the control of FDR and the beneficial power properties of GraphMM, whether the model specification is valid or violated in various ways. The methodology also reveals potentially interesting brain regions that exhibit significantly different structure between normal subjects and those suffering mild cognitive impairment.

The Dirichlet process mixture (DPM) model also entails a clustering of the inference units, with units in the same cluster block if (and only if) they share the same parameter values. The DPM model has been effective at representing heterogeneity in a system of parameters (e.g., Muller and Quintana, 2004), and in improving sensitivity in large-scale testing (e.g., Dahl and Newton, 2007; Dahl *and others*, 2008). Benefits typically come at a high computational cost, since in principle the posterior summaries require averaging over all partitions of the units (e.g., Blei and Jordan, 2006). There are also modeling costs: DPM’s usually have a product-partition form in which the likelihood function factors as a product over blocks of the partition (Hartigan, 1990). We observe that independence between blocks is violated in brain-image data in a way that may lead to inflation of the FDR.

In the present work, vertices of the graph correspond to variables in a data set and the undirected edges convey relational information about the connected variables, due to associations with the context of the data set, such as temporal, functional, spatial, or anatomical information. The graphs we consider constitute an auxiliary part of observed data. For clarity, these graphs may or may not have anything to do with undirected graphical representations of the dependence in a joint distribution (e.g., Lauritzen, 1996), as in the graphical models literature. For us, the graph serves to constrain patterns in the expected values of measurements. By limiting changes in expected values over the graph, we aim to capture low complexity of the system. An alternative way to model low-complexity is through smoothed, bandlimited signals (e.g., Ortega *and others*, 2018; Chen *and others*, 2016). Comparisons between the approaches are warranted. We have advanced the idea of latent graph-respecting partitions that constrain expected values into low-dimensional space. Figure S10 of the Supplementary material available at *Biostatistics* online investigates benefits of the graph-respecting assumption on posterior concentration and supports the treatment of this constraint as having a regularizing effect. The partition is paired with a vector of block-specific change indicators to convey the discrete part of the modeling specification. We used a uniform distribution over graph-respecting partitions in our numerical experiments and have also considered more generally the distribution found by conditioning a product-partition model (PPM) to be graph-respecting. In either case, two vertices that are nearby on the graph are more likely to share expected values, in contrast to the exchangeability inherent in most partition models. Graph restriction greatly reduces the space of partitions; we enumerated all such partitions in our proposed graph-local computations. When the generative situation is similarly graph restricted, we expect improved statistical properties; but we also showed that FDRs are controlled even if the generative situation is not graph respecting. Special cases of graph-restricted partitions have been studied by others, including Page and Quintana (2016) for lattice graphs, Caron and Doucet (2009) for decomposable graphs, and Blei and Frazier (2011) for graphs based on distance metrics. When $G$ is a complete graph, there is no restriction and all partitions have positive mass. When $G$ is a line graph, the graph-respecting partition model matches Barry and Hartigan (1992) for change-point analysis.

It is important to study resistance of the GraphMM inferences to model violations, especially as the reported empirical findings reveal a power advantage in some cases. Beyond the empirically guided simulation study (Section 3.2), we performed additional simulations to examine the effects of non-normal emissions when $G$ is a line graph. We find good FDR control for highly skewed and very heavy-tailed cases and improved properties with increasing sample size (Figures S11 and S12 of the Supplementary material available at *Biostatistics* online).

**Fig. 5:**
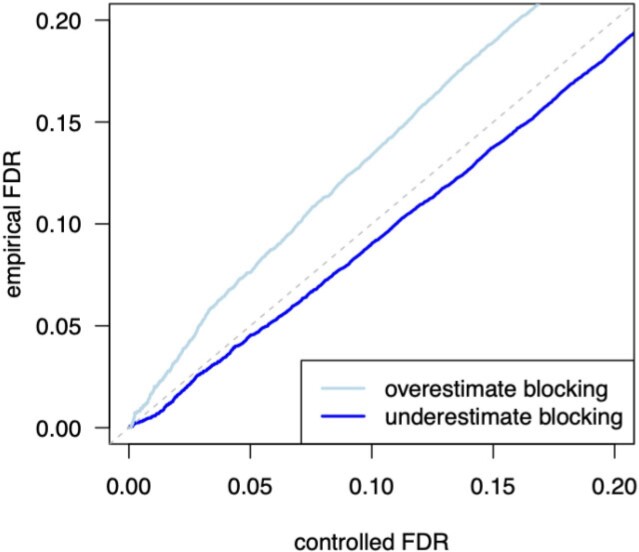
Reconsider the toy problem from Section 1, but suppose we mis-specify the blocking probability $p_{\rm block}$ (i.e., the generative value is not the same as the value we use to compute local FDR). The plot shows the empirical FDR that is realized by ranking test units via local FDR in the mis-specified model; we take one setting in which the blocking rate is assumed to be $0.1$ when it actually higher $(0.8)$ (dark blue), and a second setting that is reversed. Other parameters are as in Figure 1, except we simulate $10^6$ draws from the model to reduce variation in FDR estimates. Mis-specifying the blocking rate is not a problem here when the rate is underestimated, but we lose FDR control (light blue) if we overestimate the blocking.

There are opportunities to increase the flexibility of GraphMM, especially with regard to the induced prior distribution over graph-respecting partitions. The adopted uniform distribution on such partitions of the $3\times 3$ local lattice, for example, implies a median of four blocks. It also implies a distribution on the number of voxels in the same block as the voxel on test; for instance, there is probability $0.71$ that this central block contains no more than three voxels. Equivalently, we are insulating the test node from direct influences of data very far away. To examine the implications, we reconsider the toy example from Figure 1. Suppose that the analyst computes local FDR using an assumed $p_{\rm block}$ that is different from whatever value is generating the data. Figure 5 shows the effect on control of the FDR; in particular, if the analyst overestimates the blocking rate (i.e., over-regularizes), there is a loss of FDR control. If the blocking rate is underestimated, then this control is retained. In principle there is information in the data about the blocking rate, and future efforts could aim to take advantage in brain imaging, genomics, or other domains with graph-associated data.

## Supplementary Material

kxab001_Supplementary_Data
