# Supplementary material for: Dimension constraints improve hypothesis testing for large-scale, graph-associated, brain-image data
Source: Biostatistics. 2021 Feb 22;23(3):860–74. doi: 10.1093/biostatistics/kxab001 (PMC9295049; doi:10.1093/biostatistics/kxab001)
Supplement: kxab001_Supplementary_Data [file kxab001_supplementary_data.pdf]

Biostatistics (2021), **0**, 0, *pp.* 1–32
doi:10.1093/Biostatistics/output

Supplementary Material: Dimension constraints improve hypothesis testing for large-scale, graph-associated, brain-image data

Vo, Mishra, Ithapu, Singh & Newton

Version: January 6, 2021

This supplement is organized to match the sectioning of the main document. In summary,

1. Introduction
   - Local false discovery rate in toy example
   - Software versions
2. Methods

   2.1 Data Structure and Inference Problem
   - Graph respecting partitions

   2.2 Graph-based mixture model
   - Local subgraph calculations
   - Marginal likelihood
   - Hyperparameters
3. Results

   3.1 Brain MRI study, ADNI-2

   3.2 Data driven simulations

© The Author 2021. Published by Oxford University Press. All rights reserved. For permissions, please e-mail: journals.permissions@oup.com

- Scenarios 1-5
- Robustness to graph structure

3.3 ADNI-2 data analysis

- Number of significant voxels at various thresholds and cluster sizes
- Results from different local graphs

4. Discussion

- Compute time
- Importance of graph-respecting constraint
- Further robustness studies, line graph

Supplementary Table S1: mixture structure, toy problem; h encodes a Gaussian joint density of arguments, with mean zero in all components, marginal variance $1+\sigma^2$, and exchangeable covariance with all correlations equal to $1/(1+\sigma^2)$; fourth column indicates the number of free parameters integrated to give the last column.

Pair	Unit 1	Unit 2	mix prob	# free	predictive density
block	null	null	$p_{\text{block}}p_0$	1	$h(x_1, x_2, y_1, y_2)$
block	alt	alt	$p_{\text{block}}(1-p_0)$	2	$h(x_1, x_2)h(y_1, y_2)$
split	null	null	$(1-p_{\text{block}})p_0^2$	2	$h(x_1, y_1)h(x_2, y_2)$
split	null	alt	$(1-p_{\text{block}})p_0(1-p_0)$	3	$h(x_1, y_1)h(x_2)h(y_2)$
split	alt	null	$(1-p_{\text{block}})p_0(1-p_0)$	3	$h(x_2, y_2)h(x_1)h(y_1)$
split	alt	alt	$(1-p_{\text{block}})(1-p_0)^2$	4	$h(x_1)h(x_2)h(y_1)h(y_2)$

1. Introduction

lfdr_2 in the toy problem

On the toy problem described in Section 1 of the main paper, we assume that we have observables X_1 and X_2 from the first condition and Y_1 and Y_2 from the second. Means are μ_{X_1}, μ_{X_2}, μ_{Y_1} and μ_{Y_2} respectively, conditioned upon which, the observables are independent normals with those means and with constant variance $\sigma^2 > 0$. The four latent means fluctuate over the system by a discrete mixture that indicates strict equality constraints; the unconstrained common levels then fluctuate independently and according to the standard normal distribution. Discrete mixing is by two *coins*; the first has success probability p_{block}; in that event, called *block*, $\mu_{X_1} = \mu_{X_2}$ and $\mu_{Y_1} = \mu_{Y_2}$. Otherwise, the units are *split*. A second coin has success probability p_0; this is tossed *once* if there is blocking and *twice* if there is no blocking. Success on this second coin indicates $\mu_{X_1} = \mu_{Y_1}$ (which is the null hypothesis of interest). Supplementary Table S1 shows the six discrete outcomes, their mixing probability, as well as the joint density of the observables conditional on that mixture component, but marginal to the latent mean values themselves. From this table, the local FDR is $\text{lfdr}_2 = P(\mu_{X_1} = \mu_{Y_1} | x_1, x_2, y_1, y_2)$

$$\text{lfdr}_2 \propto p_{\text{block}}p_0 h(x_1, x_2, y_1, y_2) + (1-p_{\text{block}})(p_0^2 h(x_1, y_1)h(x_2, y_2) + (1-p_0)p_0 h(x_1, y_1)h(x_2)h(y_2))$$

with proportionality resolved by summing over the six discrete possibilities. The vertical axis of Fig. 1 (main) is the conditional FDR of the list, which, for list size n, we compute as

$$\mathrm{FDR}(n) = \frac{1}{n}\sum_{i=1}^{n} \mathrm{lfdr}(i),$$

where the $\mathrm{lfdr}(i)$ is the *ith* smallest lfdr.

Software versions

For statistical parametric and nonparametric mapping we used `SPM12` and `SnPM13`, respectively. We used `xjview8` for visualization and information to interpret significant voxels. For R packages, we used

- `stats` v 3.5.2 (for B-H adjust)
- `locfdr` v 1.1-8
- `qvalue` v 2.12.0
- `ashr` v 2.2-7

2. Methods

2.1 *Data structure and inference problem*

Graph respecting partitions: Let $G = (V, E)$ denote a simple undirected graph. Over the n nodes in V we are interested in partitions $\pi = \{b\}$, corresponding to disjoint subsets of V, (i.e., blocks), whose union equals the whole vertex set V. We say that π respects G if each block b induces a connected subgraph G_b, and we are interested in probability distributions over this restricted class of partitions as an avenue to improved statistical inference. Observe first that a graph-respecting partition π may be encoded with a vector of binary edge variables: $Z = \{Z_e : e \in E\}$ such that any two neighboring nodes in the same block have $Z_e = 1$, and any

two neighboring nodes in different blocks have $Z_e = 0$. In general, these requirements – that within-block edges are on $(Z_e = 1)$ and between-block edges are off $(Z_e = 0)$ – are such that some binary vectors Z are not allowed. One exception is when G is a tree:

Lemma 1: There is a 1-1 correspondence between graph-respecting partitions of a tree on n nodes and the set of length $n - 1$ binary vectors.

Notice that we can recover the blocks of the encoded partition by creating a new graph G' from the original G through the removal of edges where $Z_e = 0$. Then the connected components of what we call the decimated graph G' are the blocks of π. In this special case where G is a tree, we could develop MCMC proposals by randomizing the representation vector Z. But how could we take advantage of this scheme if G is not a tree?

Suppose that G is connected. (If not, we need to consider the following computation performed separately on the different connected components.) Let $\mathcal{S}$ denote the set of spanning trees of G and $Z_S = \{Z_e : e \in \text{edges}(S)\}$ be a binary edge labeling of spanning tree $S \in \mathcal{S}$. Together, the tree S and its edge labeling Z_S provide a data augmentation of the partition π. Clearly a partition can be derived from $\phi = (S, Z_S)$ by decimating the edges of G that are either not in S or are in S but with edge-label 0, and then associating blocks with the connected components of the decimated graph. In general, there will be multiple ϕ's corresponding to a given partition π, each one associated with a different spanning tree of G; we call $\Phi_\pi = \{\phi = (S, Z_S) : \pi(\phi) = \pi\}$, where we've allowed notation $\pi(\phi)$ to denote the partition induced by the input. We find a formula for the cardinality of Φ_π, which may be useful in deriving MCMC samplers against a particular target distribution over partitions.

Important: Material in this subsection describes a Markov chain Monte Carlo algorithm that could be used for `GraphMM`, but that was not used used in local posterior computations presented in the main paper. Conveniently, the sampler also allows prior sampling, which we used to enumerate the graph-respecting partitions for subsequent non-Monte-Carlo computa-

tion. We include the subsection for completeness.

For each block b of a graph-respecting partition π, the induced subgraph

$$G_b = (V_b = b, E_b = \{e = \{i,j\} : i,j \in b, e \in E\})$$

is connected and has at least one spanning tree; the total number of such trees for G_b is $N_b = \det(L_b)$, where L_b is any $n_b - 1 \times n_b - 1$ principal sub matrix of the graph Laplacian $D_b - A_b$, where D_b is a diagonal matrix holding the node degrees in G_b, and A_b is the incidence matrix describing this same subgraph. This is an application of Kirchhoff's matrix-tree theorem. Of importance in relating the different subgraphs G_b is the hyper-graph H in which nodes are blocks of π and multi-edges between nodes correspond to all the between-block edges in G:

$$H = \left(V_H = \pi, E_H = E \cap [\cup_b E_b]^c\right).$$

By another application of the matrix-tree theorem, the number N_H of spanning trees of H is $\det(L_H)$, where L_H is an $n_H - 1 \times n_H - 1$ principal sub matrix of $D_H - A_H$.

Lemma 2: The cardinality of Φ_π, denoted $N(\pi)$, satisfies $N(\pi) = N_H \prod_{b \in \pi} N_b$.

We augment the partition π of G to the pair $\phi = (S, Z_S)$ holding both a spanning tree S and a vector of binary labels Z_S which encodes the partition of S. For any target distribution $p(\pi)$ we develop a Markov chain sampler by running a chain over the space of ϕ's, which by construction is a union of sets Φ_π. The idea is that the target distribution of a Markov chain in the augmented space is

$$p(\phi) = p(\pi(\phi)) \times \frac{1}{N[\pi(\phi)]}. \tag{2.1}$$

Generatively, this is equivalent to realizing π from $p(\pi)$ and then selecting one of its $N(\pi)$ representations $\phi \in \Phi_\pi$ uniformly at random. Of course we cannot easily generate from $p(\pi)$; the point is that the data augmentation offers a variety of proposal mechanisms that might drive a Metropolis-Hastings sampler. We envision two coupled proposals:

1. Update Z_S for a fixed spanning tree (and thus change the partition).

2. Update spanning tree S but keep the same partition $\pi(\phi)$ (and thus update Z_S).

Partition update: Suppose the chain is at state $\phi = (S, Z_S)$ and we propose a new state $\phi^* = (S, Z_S^*)$ by randomizing the representation vector Z_S but keeping the spanning tree fixed. For example, we could generate entries Z_e^* as independent Bernoulli trials with some edge-specific success probability. Data dependent probabilities might improve mixing, and we might randomize only a fraction of the entries in order to simplify the update and improve its acceptance rate. Call $q_S(Z_S^*|Z_S)$ the probability mass associated with this proposal; then the Metropolis-Hastings ratio for this case is

$$\begin{aligned}\text{MH} &= \frac{p(\phi^*)}{p(\phi)} \times \frac{q_S(Z_S|Z_S^*)}{q_S(Z_S^*|Z_S)} \\ &= \frac{p[\pi(\phi^*)]}{p[\pi(\phi)]} \times \frac{N[\pi(\phi)]}{N[\pi(\phi^*)]} \times \frac{q_S(Z_S|Z_S^*)}{q_S(Z_S^*|Z_S)}.\end{aligned}$$

The first ratio on the right may be relatively simple, especially for product-partition models, since only the blocks incurring some change are retained. The third ratio, of q's, may also be simple if we use independent Bernoulli's to randomize the entries. What is critical then is the middle ratio, whose value is available according to Lemma 2.

Tree update: Suppose the chain is at state $\phi = (S, Z_S)$ and we propose a new spanning tree S^* uniformly over the set of possibilities $\mathcal{S}$, for example via Wilson's loop-erased random walk. Relative to the current partition $\pi = \pi(\phi)$, there is exactly one binary encoding vector $Z_{S^*}^*$ corresponding to S^*, by Lemma 1, and so we have proposed the state $\phi^* = \left(S^*, Z_{S^*}^*\right)$ that induces the same partition, and thus is an element of Φ_π. The Metropolis-Hastings ratio for target (2.1) is

$$\text{MH} = \frac{p(\phi^*)}{p(\phi)} \times \frac{p(\phi^* \longrightarrow \phi)}{p(\phi \longrightarrow \phi^*)} = 1 \times 1.$$

Thus, any proposal obtained by randomizing the spanning tree while keeping partition fixed is surely accepted.

The spanning tree representation above could be used to construct a posterior MCMC sampler. We experimented with such a sampler, but the main paper reports local computations based on explicit sumations over the discrete states; ordinary MCMC was not required. However, we did use a prior-sampler version of the spanning-tree sampler as a simple way to enumerate all graph respecting partitions on a given local graph.

2.2 *Graph-based mixture model*

Local subgraph calculations: We do not apply the model of Section 2 (main) on the entire graph, due to complexity and dimensionality. We expect most of the information relevant to inference about one node to come through nearby nodes, so we deploy local graph calculations amenable to parallel computing. For each node v, consider a local subgraph $\mathcal{N}_v$ containing node v. $\mathcal{N}_v$ is chosen such that it is a connected component of graph G. `GraphMM`, then, is applied to model the data on this subgraph $(\boldsymbol{X}_{\mathcal{N}_v}, \boldsymbol{Y}_{\mathcal{N}_v}) := \{(\boldsymbol{X}_u, \boldsymbol{Y}_u) : u \in \mathcal{N}_v\}$ The node-specific posterior probabilities are

$$l_v := P(H_{0,v}|\mathbf{X}_{\mathcal{N}_v}, \mathbf{Y}_{\mathcal{N}_v}) = \sum_{\Psi,\mathbf{\Delta}} \mathbb{1}(\Delta_k = 0)1(v \in b_k)P(\mathbf{\Delta}, \Psi|\mathbf{X}_{\mathcal{N}_v}, \mathbf{Y}_{\mathcal{N}_v}) \tag{2.2}$$

The local procedure is illustrated by Fig. S1.

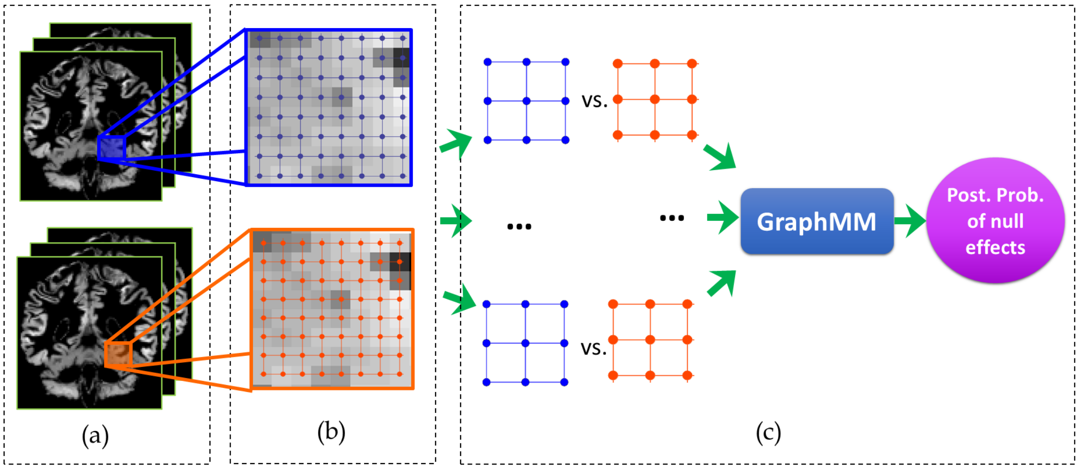

Supplementary Figure S1: Local subgraph calculations. (a) shows pre-processed MRI images of two conditions. (b) shows lattice graphs associated with the data. (c) shows local procedure, in which local subgraph $\mathcal{N}_v$ includes v and eight adjacent nodes. GraphMM model is applied to the subgraph data to get posterior probability of null effects l_v as in (2.2).

Algorithm 1 Local subgraph calculation

Input: pre-processed MRI data $\boldsymbol{X}$, $\boldsymbol{Y}$.

Output: per voxel posterior probability of differential structure.

procedure GraphMM($\boldsymbol{X}$, $\boldsymbol{Y}$)
  for v in nodes **do**
    $G_{\text{local}} \leftarrow$ local graph around v
    $(\boldsymbol{X}_{\mathcal{N}_v}, \boldsymbol{Y}_{\mathcal{N}_v}) \leftarrow$ local data around v
    for $(\Psi, \boldsymbol{\Delta})$ in graph-respecting partitions of G_{local} **do**
      $p(\boldsymbol{X}_{\mathcal{N}_v}, \boldsymbol{Y}_{\mathcal{N}_v} | \Psi, \boldsymbol{\Delta}) \leftarrow$ marginal density of data local to v
      $p(\Psi, \boldsymbol{\Delta}) \leftarrow$ prior mass of local state.
    end for
    Scale to get $p(\Psi, \boldsymbol{\Delta} | \boldsymbol{X}_{\mathcal{N}_v}, \boldsymbol{Y}_{\mathcal{N}_v})$ for all graph-respecting partitons $(\Psi, \boldsymbol{\Delta})$
    $l_v \leftarrow P(H_{0,v} | \boldsymbol{X}_{\mathcal{N}_v}, \boldsymbol{Y}_{\mathcal{N}_v})$ # Using formula 2.2
  end for
end procedure

For results in Sections 3.1 and 3.2 (main), we did local approximation on a 3×3 neighborhood of each node, as illustrated in Fig. S1. In this case we can enumerate all the graph-respecting partitions (we devised a data-augmentation sampling scheme that makes use of spanning trees within the input graph; see Supplementary section 2.1). Then, we are able to enumerate all the pairs $(\Psi, \boldsymbol{\Delta})$ and compute the exact posterior distribution. In addition to the 3×3 local, within-slice, subgraph, we used two other local star subgraphs in the calculations reported in Figure 9.

Marginal likelihood: We derive the marginal likelihood using Laplace approximation. Consider the notations as in Section 2 of main paper. Let K_{Ψ} be the number of blocks corresponding to partition $\boldsymbol{\Psi}$ and K_{diff} be the number of changed blocks, which means

$$K_{\text{diff}} = \sum_{k=1}^{K_{\Psi}} \mathbb{1}(\Delta_k = 1)$$

Denote the ordered indices of changed blocks as $(j_1, j_2, \ldots, j_{K_{\text{diff}}})$. We re-parametrize the model in order to remove the clustering constraints on the means

$$\boldsymbol{\epsilon} := (\delta_{j_1}, \delta_{j_2}, \ldots, \delta_{j_{K_{\text{diff}}}})$$

Then, the free parameters are $(\boldsymbol{\varphi}, \boldsymbol{\epsilon})$ and the marginal likelihood function can be written as

$$\begin{aligned} f(\boldsymbol{X}, \boldsymbol{Y} | \boldsymbol{\Psi}, \boldsymbol{\Delta}) &= \int f(\boldsymbol{X}, \boldsymbol{Y}, \boldsymbol{\varphi}, \boldsymbol{\epsilon} | \boldsymbol{\Psi}, \boldsymbol{\Delta}) \mathrm{dP}(\boldsymbol{\varphi}) \mathrm{dP}(\boldsymbol{\epsilon}) \\ &= C_1 C_2 \int_{\mathbb{R}^{K_{\Psi}+K_{\text{diff}}}} \exp\left[(\mathrm{df} + M_X) F(\boldsymbol{\varphi}, \boldsymbol{\epsilon}) \right] \mathrm{d}\boldsymbol{\varphi}\, \mathrm{d}\boldsymbol{\epsilon} \end{aligned}$$

where

$$C_1 = C_1(N, M_X, M_Y, \mathrm{df}) = \frac{(|A||B|)^{\frac{d}{2}} \Gamma_N \left(\frac{\mathrm{df}+M_X}{2} \right) \Gamma_N \left(\frac{\mathrm{df}+M_Y}{2} \right)}{\left[\Gamma_N \left(\frac{\mathrm{df}}{2} \right) \right]^2}$$

$$C_2 = C_2(\boldsymbol{\Psi}, \boldsymbol{\Delta}, \tau, \sigma) = \exp\left[-K_{\Psi} \log(\tau\sqrt{2\pi}) - K_{\text{diff}} \log(\sigma\sqrt{2\pi}) \right]$$

$$F(\boldsymbol{\varphi}, \boldsymbol{\delta}) = -\frac{1}{2}\log|\widetilde{A}| - \frac{\text{df} + M_Y}{2(\text{df} + M_X)}\log|\widetilde{B}| - \frac{1}{2\tau^2(\text{df} + M_X)}\sum_{k=1}^{K_\Psi}(\varphi_k - \mu_0)^2$$
$$-\frac{1}{2\sigma^2(\text{df} + M_X)}\sum_{l=1}^{K_{\text{diff}}}(\epsilon_l - \delta_0)^2$$

$$\widetilde{A} = A + (M_X - 1)S_1 + M_X S_2$$
$$S_1 = \frac{1}{M_X - 1}\sum_{m=1}^{M_X}(\boldsymbol{X_m} - \overline{\boldsymbol{X}})(\boldsymbol{X_m} - \overline{\boldsymbol{X}})^\tau$$
$$S_2 = (\overline{\boldsymbol{X}} - \boldsymbol{\mu_X})(\overline{\boldsymbol{X}} - \boldsymbol{\mu_X})^\tau$$

$$\widetilde{B} = B + (M_Y - 1)T_1 + M_Y T_2$$
$$T_1 = \frac{1}{M_Y - 1}\sum_{r=1}^{M_Y}(\boldsymbol{Y_r} - \overline{\boldsymbol{Y}})(\boldsymbol{Y_r} - \overline{\boldsymbol{Y}})^\tau$$
$$T_2 = (\overline{\boldsymbol{Y}} - \boldsymbol{\mu_Y})(\overline{\boldsymbol{Y}} - \boldsymbol{\mu_Y})^\tau$$

Applying Laplace's approximation, we get

$$\log f(\boldsymbol{X}, \boldsymbol{Y}|\boldsymbol{\Psi}, \boldsymbol{\Delta}) \approx \log C_1 + \log C_2 + \frac{K_\Psi + K_{\text{diff}}}{2}\log\frac{2\pi}{\text{df} + M_X}$$
$$+(\text{df} + M_X)F(\widehat{\boldsymbol{\varphi}}, \widehat{\boldsymbol{\epsilon}}) - \frac{1}{2}\log|-H(F)(\widehat{\boldsymbol{\varphi}}, \widehat{\boldsymbol{\epsilon}})| \tag{2.3}$$

In the next step, we derive the explicit formula for the gradient and Hessian matrix of F. Let L be the allocation matrix with size $N \times K_\Psi$ where $c_{vk} = 1$ if and only iff node v belong to block k. Let R be a $K_\Psi \times K_{\text{diff}}$ matrix such that column lth of R has value 1 at position j_l and has value 0 at other postions. Then we can relate the mean vectors with the new parameters $(\boldsymbol{\varphi}, \boldsymbol{\epsilon})$ as follows

$$\boldsymbol{\delta} = R\boldsymbol{\epsilon}$$

$$\boldsymbol{\mu_X} = L\boldsymbol{\varphi} \qquad \boldsymbol{\mu_Y} = L(\boldsymbol{\varphi} + \boldsymbol{\delta})$$

We consider following notations.

$$S_3 = \frac{1}{M_X - 1}A + S_1 \qquad T_3 = \frac{1}{M_Y - 1}B + T_1$$

$$v_X = S_3^{-1}\overline{\boldsymbol{X}} \qquad v_Y = T_3^{-1}\overline{\boldsymbol{Y}}$$

$$s_0 = \frac{M_X - 1}{M_X} + \overline{\boldsymbol{X}}^\tau v_X \qquad t_0 = \frac{M_Y - 1}{M_Y} + \overline{\boldsymbol{Y}}^\tau v_Y$$

$$Q_X = L^\tau S_3^{-1} L \qquad Q_Y = L^\tau T_3^{-1} L$$

$$w_X = S_3^{-1}\boldsymbol{\mu_X} \qquad w_Y = T_3^{-1}\boldsymbol{\mu_Y}$$

$$u_X = L^\tau(w_X - v_X) \qquad u_Y = L^\tau(w_Y - v_Y)$$

$$b_X = \frac{1}{s_0 - 2{v_X}^\tau \boldsymbol{\mu_X} + \boldsymbol{\mu_X}^\tau w_X}$$

$$b_Y = \frac{1}{t_0 - 2{v_Y}^\tau \boldsymbol{\mu_Y} + \boldsymbol{\mu_Y}^\tau w_Y}$$

The formula for the gradient of F is.

$$\frac{\partial F}{\partial \boldsymbol{\varphi}} = \left[-b_X u_X - \frac{\mathrm{df} + M_Y}{\mathrm{df} + M_X} b_Y u_Y - \frac{1}{\tau^2(\mathrm{df} + M_X)}(\varphi_k - \mu_0 \boldsymbol{J}_{K_\Psi})\right]^\tau$$

$$\frac{\partial F}{\partial \boldsymbol{\epsilon}} = \left[-\frac{\mathrm{df} + M_Y}{\mathrm{df} + M_X} b_Y u_Y - \frac{1}{\sigma^2(\mathrm{df} + M_X)}(\delta_k - \delta_0 \boldsymbol{J}_{K_\Psi})\right]^\tau R$$

$$\frac{\partial F}{\partial(\boldsymbol{\varphi}, \boldsymbol{\epsilon})} = \begin{bmatrix} \frac{\partial F}{\partial \boldsymbol{\varphi}} & \frac{\partial F}{\partial \boldsymbol{\epsilon}} \end{bmatrix}$$

where $\boldsymbol{J}_{K_\Psi}$ is a vector of ones with size K_Ψ.

Next, the formula for Hessian matrix of F is

$$\frac{\partial^2 F}{\partial \boldsymbol{\varphi}\, \partial \boldsymbol{\varphi}^\tau} = -b_X(Q_X - 2b_X u_X {u_X}^\tau) - \frac{\mathrm{df} + M_Y}{\mathrm{df} + M_X} b_Y (Q_Y - 2b_Y u_Y {u_Y}^\tau) - \frac{1}{\tau^2(\mathrm{df} + M_X)} \mathbb{1}_{K_\Psi \times K_\Psi}$$

$$\frac{\partial^2 F}{\partial \boldsymbol{\epsilon}\, \partial \boldsymbol{\epsilon}^\tau} = R^\tau \left[-\frac{\mathrm{df} + M_Y}{\mathrm{df} + M_X} b_Y (Q_Y - 2b_Y u_Y {u_Y}^\tau)\right] R - \frac{1}{\sigma^2(\mathrm{df} + M_X)} \mathbb{1}_{K_{\mathrm{diff}} \times K_{\mathrm{diff}}}$$

where $\mathbb{1}_{K \times K}$ is the identity matrix of size $K \times K$.

$$\frac{\partial^2 F}{\partial \boldsymbol{\varphi}\, \partial \boldsymbol{\epsilon}^\tau} = \left[-\frac{\mathrm{df} + M_Y}{\mathrm{df} + M_X} b_Y (Q_Y - 2b_Y u_Y {u_Y}^\tau)\right] R$$

$$H(F) = \begin{bmatrix} \frac{\partial^2 F}{\partial \boldsymbol{\varphi}\, \partial \boldsymbol{\varphi}^\tau} & \frac{\partial^2 F}{\partial \boldsymbol{\varphi}\, \partial \boldsymbol{\epsilon}^\tau} \\ \frac{\partial^2 F}{\partial \boldsymbol{\epsilon}\, \partial \boldsymbol{\varphi}^\tau} & \frac{\partial^2 F}{\partial \boldsymbol{\epsilon}\, \partial \boldsymbol{\epsilon}^\tau} \end{bmatrix}$$

Finally, the maximizer $((\widehat{\boldsymbol{\varphi}}, \widehat{\boldsymbol{\epsilon}}))$ can be found using Broyden–Fletcher–Goldfarb–Shanno algorithm.

We point out that the calculation above takes sample covariance matrices $S1$ and $T1$, adjusts them by adding a scaled hyper-parameter covariance matrix (A or B): $S_3 = \frac{1}{M_X - 1}A + S_1$, and $T_3 = \frac{1}{M_Y - 1}B + T_1$ where M_X and M_Y are the sample size of group1 and group 2 respectively. We require these adjusted covariance matrices to be of full rank in order to deploy calculations above. This has not been difficult in the context of local graph computations where the dimensionality is low compared to sample size.

Hyperparameters: The analysis of `GraphMM` involves estimating hyper parameters: prior null probability p_0, prior mean μ_0 and standard deviation τ^2 of block mean of group 1, prior mean δ_0 and standard deviation σ^2 of difference in block mean between 2 groups, prior covariance matrix A for group 1 and matrix B for group 2. Different strategies for estimating hyperparameters have been considered,

- *Estimating prior null probability p_0:* We experimented with both `qvalue` or `ahsr` to get the estimated value of p_0. Package `qvalue` produces conservative estimate of p_0 without any assumption on the distribution of effects. Hence it is a safe and conservative choice under general settings. Package `ashr`, on the other hand, provides conservative estimate under the assumption that the distribution of effects is unimodal. Furthermore, in our graph-based mixture model, the distribution of effects δ_k was assumed to be a mixture of probability mass at 0 and normal distribution, which satisfies unimodal assumption. Therefore, using package `ashr` to estimate for p_0 meshes with our `GraphMM`. The estimation of p_0 is based on the whole dataset, computing prior to the local approximation procedure. In reported computations we used `ashr` package for p_0.

- *Estimating other hyperparameters:* We consider 3 approaches: global, local and mixed es-

timation. With global estimation, the hyperparameters are estimated using the whole dataset and computed prior to the local approximation procedure. With local estimation, hyperparameters are estimated for each neighborhood, during the local approximation procedure. With mixed estimation, all hyperparameters are estimated locally except for matrices A and B, which are estimated globally. These approaches, local, mixed and global provides increasingly conservative estimates in that order. In the brain simulation and application, we present results using mixed estimation.

3. Results

3.1 *Brain MRI study: ADNI-2*

The Alzheimer's Disease Neuroimaging (ADNI) project was launched in 2003 by the National Institute on Aging, the National Institute of Biomedical Imaging and Bioengineering, the Food and Drug Administration, private pharmaceutical companies, and nonprofit organizations. The overarching goal of ADNI study comprises of detecting Alzheimer's Disease (AD) at the earliest possible stage, identifying ways to track the disease progression with biomarkers and support advances in AD intervention, prevention and treatments. ADNI is the result of the efforts of many co-investigators from a broad range of academic institutions and private corporations, and subjects have been recruited from over 50 sites across the U.S. and Canada.

3.2 *Data-driven simulations*

Scenarios 1-5: Figure S2 and Algorithm 2 show the general structure of the empirically-guided simulation study that is varied in 5 specific scenarios (Tables S2 and S3). Three data sets are drawn in each scenario. Simulations are of data on a single coronal MRI slice.

For constructing the graph-respecting partition, let us explain step 6 more carefully. We construct a "meta dataset" : data at each node is a vector of following components: (data values

Algorithm 2 General framework for all the simulation scenarios

Input: MRI dataset for condition 1, **G1**, a $N \times M_X$ matrix; real dataset for condition 2, **G2**, a $N \times M_Y$ matrix.
Output: synthetic dataset 1 $\boldsymbol{X}$; synthetic dataset 2 $\boldsymbol{Y}$.

Step 1: $S_1 \leftarrow$ sample-covariance(**G1**).
Step 2: $S_2 \leftarrow$ sample-covariance(**G2**).
Step 3: $\mathbf{V} \leftarrow S_1 + 0.5\mathbf{I}$ # $\mathbf{I}$ is identity matrix.
Step 4: $\mathbf{W} \leftarrow S_2 + 0.5\mathbf{I}$ # Add a small value to the diagonal of S_1, S_2 to get positive definite matrices.
Step 5: $Avg \leftarrow$ average-over-replicate(**G1**, **G2**) # Avg is a vector of length N

Implement for step 6 to 9 depends on specific scenario
Step 6: $\Psi \leftarrow$ cluster(Avg) # $\Psi = \{b_1, \ldots, b_K\}$ is a graph-respecting partition on Avg
Step 7: $\Delta \leftarrow$ changed-block indicator # $\Delta = \{\Delta_1, ..., \Delta_K\}$ is a binary vector, $\Delta_k = 1$ iff b_k is a changed-block.
Step 8 & 9:
$\varphi \leftarrow$ simulated block means for condition 1
$\delta \leftarrow$ simulated changed effects # $\delta_k = 0$ iff $\Delta_k = 0$; when $\Delta_k \neq 0$, δ_k is simulated from some distribution (e.g beta, uniform)
$\nu \leftarrow \phi + \delta$ # ν is simulated block means for condition 2.
$\mu_X \leftarrow$ simulated node means for condition 1
$\mu_Y \leftarrow$ simulated node means for condition 2 # μ_X, μ_Y satisfy clustering constraints on the means w.r.t Ψ.

##########
Step 10: $\boldsymbol{X} \leftarrow$ Multivariate Normal ($\mu_X, \mathbf{V}$)
Step 11: $\boldsymbol{Y} \leftarrow$ Multivariate Normal ($\mu_Y, \mathbf{W}$)

from group 1, data values from group 2, α*x-coordinate, α*y-coordinate). Here, α is a scale parameter. Use the "greedy clustering" method to cluster the meta dataset (Collins, 2014, `http://pages.cs.wisc.edu~mcollins/software/greedy-clustering.html`). This method performs clustering based on Euclidean similarity. So, two nodes are in the same block when they are close in terms of both data values and xy-coordinates. If α is large, it would increase the weight of xy-coordinates in the clustering result. Adding xy-coordinates causes the block to be more likely to respect the lattice. Let's call this partition as P1. No graph structure is involved in this step yet. Next, consider a lattice graph associated with the data. Loop over each block of P1, check whether the block is graph-respecting (do this using igraph package to count the number of connected components). If the block isn't graph-respecting, split that block into multiple blocks so that each extra block is a connected component. In the end, each block would be a connected component of the lattice graph, hence the final partition (P2) is graph-respecting.

Supplementary Table S2: Description for simulation 1, 2 and 3. Text with blue color and figures emphasizes that these simulations differ in the average size of latent blocks. In the figures, area with magenta color shows changed-blocks. We can see that the size of changed-blocks decreases in simulation 1, 2 and 3. Especially simulation 3 has no clustering effect, i.e the block size is 1 for all blocks.

	Scenario 1	Scenario 2	Scenario 3
Step 6	* Use greedy clustering method<br>* Partition is adjusted to respect lattice graph.<br>* There are 1313 blocks, average block size is 3.9	Same as scenario 1	* Each node itself is a block.<br>* There are 5236 blocks, block size is 1
Step 7	* 50 blocks with size from 12 to 14 are chosen to be changed-block<br>* Average size of changed-block is 13.6<br>* Percentage of changed-nodes: 14.3%	* 300 blocks with size from 2 to 5 are chosen to be changed-block<br>* Average size of changed-block is 2.6<br>* Percentage of changed-nodes: 14.9%	* 15% of the nodes are chosen to be changed-nodes
Step 8, 9	$m_x \leftarrow$ block average of MRI data group 1<br>$m_y \leftarrow$ block average of MRI data group 2<br>$max.d \leftarrow \max(m_y - m_x)$<br>$min.d \leftarrow \min(m_y - m_x)$<br>$\varphi \leftarrow m_x$<br>For changed-blocks: $\delta \sim$ Uniform$(min.d, max.d)$	Same as scenario 1	$m_x \leftarrow$ block average of MRI data group 1<br>$sd_x \leftarrow$ sample block standard deviation group 1<br>$mar.m \leftarrow$ mean(m_x)<br>$mar.sd \leftarrow$ mean(sd_x)<br>$\varphi \sim$ Normal$(mar.m, mar.sd)$<br>$m_y \leftarrow$ block average of MRI data group 2<br>$max.d \leftarrow \max(m_y - m_x)$<br>$min.d \leftarrow \min(m_y - m_x)$<br>For changed-blocks: $\delta \sim$ Beta(2, 2)<br>$\delta \leftarrow \delta * (max.d - min.d) + min.d$
Figure	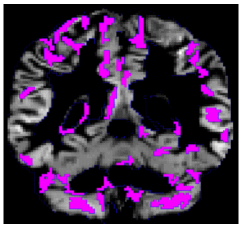	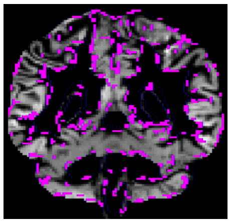	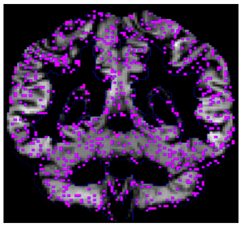

The graph-respecting partition P2 is used for both groups.

Robustness to graph structure: We follow the simulation algorithm in Figure S2, except we modify the clustering so that blocks may not be graph-respecting and also shifts between conditions may not be constant shifts on whole blocks. In Step 6, we construct a meta dataset for each group 1 and 2. we use greedy clustering to get two separate partitions (P and Q), for the two

Supplementary Table S3: Description for simulation 4 and 5. Text with blue color and figures emphasizes that these simulations differ in the percentage of changed-nodes. The figures show histogram of block avergage shifts across 2 groups for all blocks (red area) and for changed-blocks (green area).

	Simulation 4	Simulation 5
Step 6	* Use greedy clustering method<br>* Partition is adjusted to respect lattice graph.<br>* There are 1313 blocks, average block size is 3.9	Same as Simulation 4
Step 7	$m_x \leftarrow$ block average of MRI data group 1<br>$m_y \leftarrow$ block average of MRI data group 2<br>$diff \leftarrow m_y - m_x$<br>$prob \leftarrow$ increasing function of $\|diff\|$ and belongs in (0,1)<br>$\Delta \sim$ Bernoulli($prob$)<br>* Percentage of changed-nodes: 16.4%	* Similar to simulation 4, except that<br>* Percentage of changed-nodes: 50.3%
Step 8 & 9	* If block k is a changed-block:<br>$\varphi[k] \leftarrow m_x[k]$<br>$\delta[k] \leftarrow diff[k]$<br>* If block k is not a changed-block:<br>$\varphi[k] \leftarrow (m_x[k] + m_y[k])/2$<br>$\delta[k] \leftarrow 0$	Same as Simulation 4
Figure	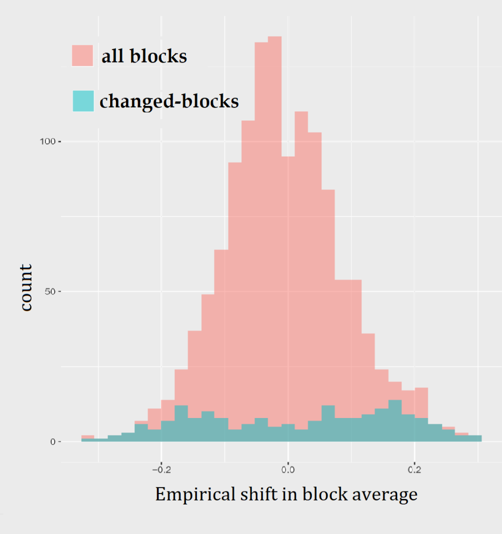	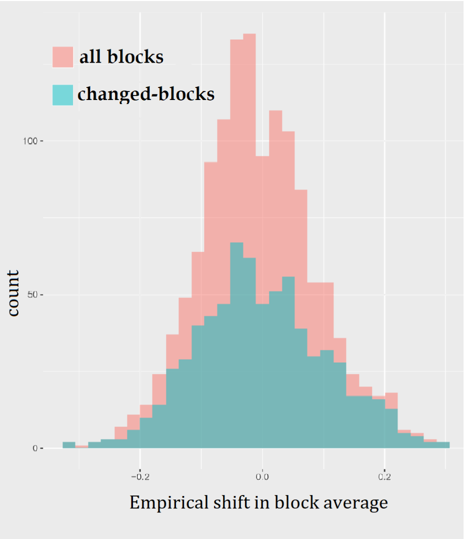

groups. So, P and Q are not graph-respecting when we downweight the spatial coordinates, and they're also different for 2 groups. We use data for each group to get the block mean for each block of P and Q. Next we "merge" a block of P with a block Q when their block means are sufficiently close: For group1, consider block 1 of P, named Pb1. For group 2, the nodes in Pb1 block would belong to several blocks of Q, say Qb1, Qb3, Qb7. Let's assume that the mean of Pb1 and Qb7 are very close, then we can "merge" Pb1 and Qb7 in the sense that the nodes in Pb1 (group1) and nodes in Qb7 (group2) have the same mean (and thus are null). After this merging step, the partitions for 2 groups are still different, each of them are also not graph-respecting. The latent mean vectors are low dimensional, but they are not as spatially coherent as in the model, and they are not consistent between groups. Other aspects of the sampling model are retained.

3.3 *ADNI-2 data analysis*

Number of significant voxels at various thresholds and cluster sizes: Expanding the analysis of Section 3.2 (main), Figure S4 presents the number of significant voxels at various FDR thresholds; Figure S5 shows sizes of clusters of significant voxels. In both cases we consider the 3D brain lattice after calculations use the 2d 3×3 local graph.

Results from different local graphs Figure S8 considers the full brain ADNI-2 example on two different `GraphMM` computations, which differ by the nature of the local graph used. In one case we use the 3×3 2-d slice graph; the second case uses the 3-d star graph centered. See Figure S9 on the marginals prior to ranking.

4. Discussion

Compute time:

The prototype GraphMM code is not especially fast. For the 3-d star-local-graph whole-brain calculation, we broke down the calculation into 465 batches of 1000 local calculations using the HT Condor parallel computing system, where each batch requested a compute node with 2GB memory and 2GB of disk. The average CPU time for each condor node was 8 hours 14 mins (min- 4hr:46min, max- 14:28) Each condor node was computing 1000 vertices. So, that's 32 sec per vertex to resolve the local subgraph computation. The lattice calculation was slower as it accounted for the greater number of graph-respecting partitions. In the main simulations, we configured HT Condor differently than above and completed a single-slice (5236 voxels) in about 2 hours of clock time.

Importance of the graph-respecting constraint:

We do a sanity check to confirm that statistical efficiency gains may arise by regularizing the expected values through the graph-respecting assumption. In a predictive simulation of the 3×3 lattice, we generate synthetic Gaussian data D as follows: expected values are guided by some generative graph-respecting partition Ψ^* (drawn from a prior); block-specific means are realized as i.i.d. Gaussian$(0, \sigma^2 = 1/4)$ variables; the 9 data elements in D deviate from these means by realizations of i.i.d. Gaussian$(0, \sigma^2 = 1)$ variables. Each simulated data set is one instance of data when the generative setting is graph-respecting. We take each such simulated data set and work out what two different analysts would surmise about the generative partition. Analyst A knows that the expected values follow some partition structure. Analyst B knows also that the expected values follow a graph-respecting partition. Each analyst computes a posterior distribution, say $P_{\text{analyst}}(\Psi|D)$, over the set of partitions; indeed each posterior dis-

tribution is concentrated at some level around the generative partition Ψ^*. A simple measure of the concentration is through the induced distribution on the Adjusted Rand Index, which measures a similarity $S(\Psi, \Psi^*)$ between two partitions. For any level of similarity, s, each analyst has a posterior probability $p_{\text{analyst}}(s, D) = P\left[S(\Psi, \Psi^*) \leqslant s|D\right]$. Figure S10 compares analysts by the average of these posterior similarity distributions $p_{\text{analyst}}(s, D)$ over data sets D. It reveals that by enforcing regularity on the prior distribution over partitions (i.e., by enforcing the graph-respecting property), we tend to place greater posterior probability mass near the generative partition. In applications where the graph conveys structure of expected values, the graph-respecting assumption may usefully regularize the local FDR computations to benefit sensitivity.

Additional simulations on line graph:

To further investigate resistance to violation of model assumptions, we simulated the `GraphMM` generative model, except we altered the emission distribution on measurements to allow heavy-tailed or skewed data. We used a line graph with 1000 nodes, and thus all internal nodes have exactly 2 neighbors. To set latent blocks, we applied a fair coin toss to each graph edge. Then we applied a further fair coin to each block to decide if it null or not. The mean value in both artificial groups at a null node was zero, and the two means values at any non-null block were draws from a normal distribution with mean zero and standard deviation $\delta = 1/4$. Supplementary Figures S11 and S12 consider two different sample size cases; the first having $n = 50$ samples per group and the second having $n = 100$ samples per group. Data were generated either standard normal deviations from groups means (top panels) or heavy-tailed or highly skewed. For the heavy-tailed case, we took the normal measurements and cubed them. For the skewed case we added standard exponential variates to the means. The central column of both figures shows normal qq-plots of the data from one group. We see generally good FDR control,

when plotting the `GraphMM` local FDR against the computed false discovery proportion on 10 simulated data sets; some lack control emerges at low FDR, especially in the heavy-tailed case. Naturally, there is some reduction in sensitivity outside the model (third column), but overall good properties. Figure S12 has double the sample size, and shows improved performance.

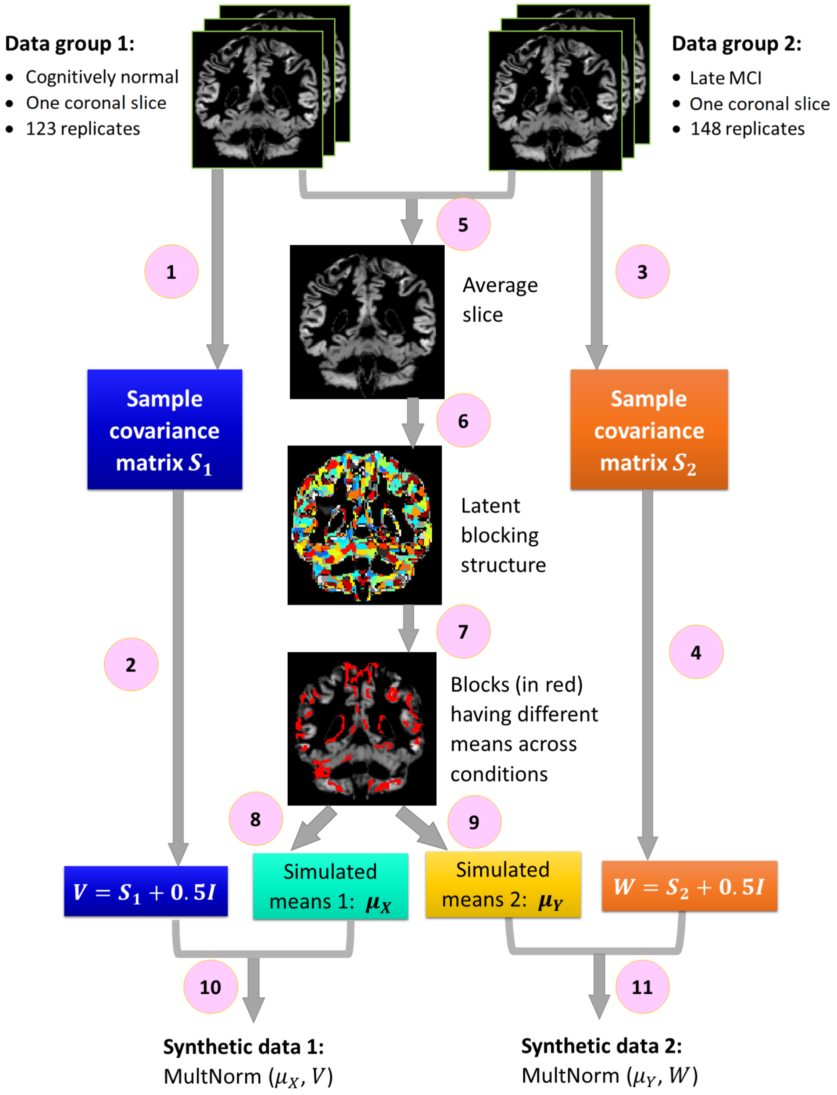

Supplementary Figure S2: Structure of data-driven simulation (Scenarios 1-5): Steps 1-4 make the correlation structure of synthetic data similar to that of MRI data. Steps 5-7 aim to mimic the mean structure and clustering pattern of MRI data. Steps 8-11 simulate data following multivariate normal distribution with specified correlation and mean structure.

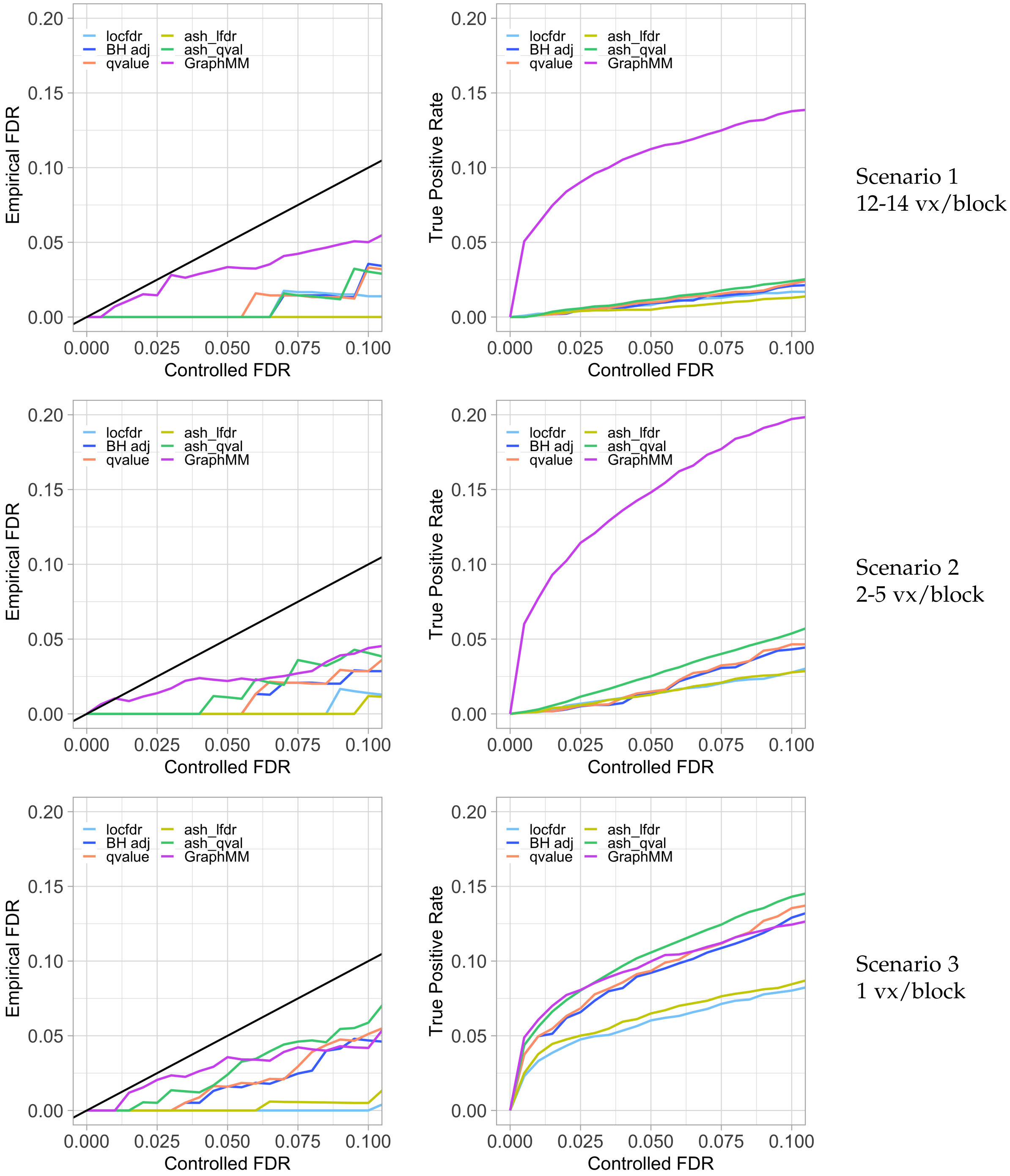

Supplementary Figure S3: Operating characteristics on synthetic data. Rows correspond to simulation scenarios (1=top, large blocks; 2=middle, small blocks; 3=bottom, tiny blocks). On the left we compare the empirical FDR with the target controlling FDR. Dominance by the diagonal (black) confirms that all methods are controlling FDR at the target rates. The right panels show how well different methods identify voxels that are truly different between the two groups. Substantial power gains are evident by GraphMM. Supplementary Table S1 provides simulation details. Top row populates Fig. 3 of main paper.

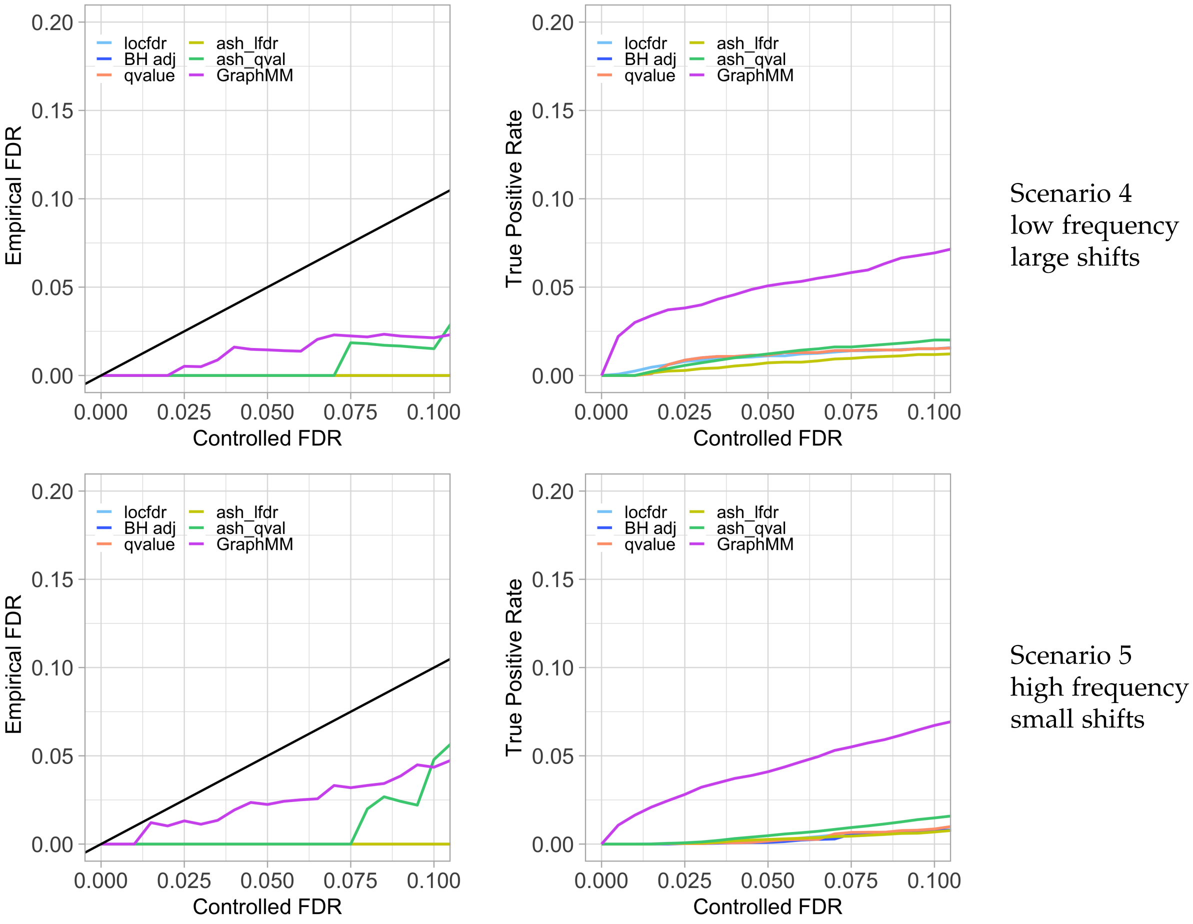

Supplementary Figure S4: Operating characteristics on synthetic data which explore different distributions of effects (Scenarios 4 and 5). Supplementary Table S2 has details.

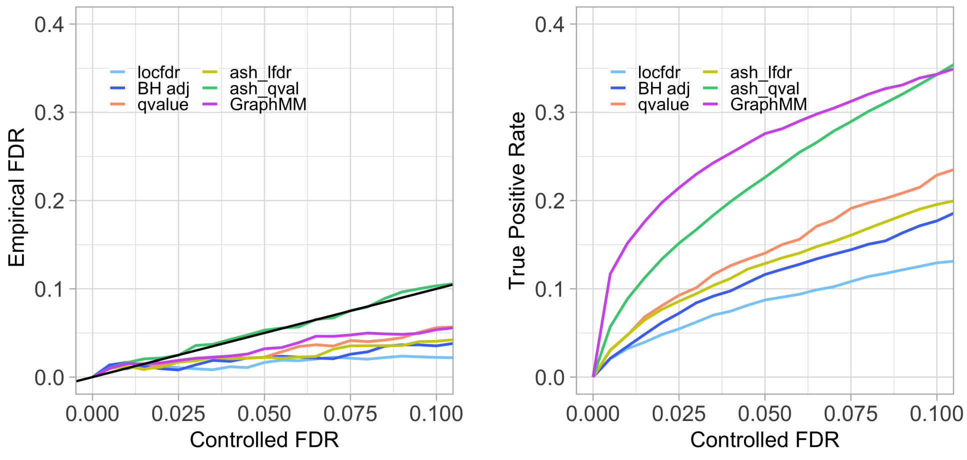

Supplementary Figure S5: Robustness to graph-respecting assumption. FDR (left) and sensitivity (right) in a case where latent partitions are not graph respecting, but have similarity 0.48 (Rand index).

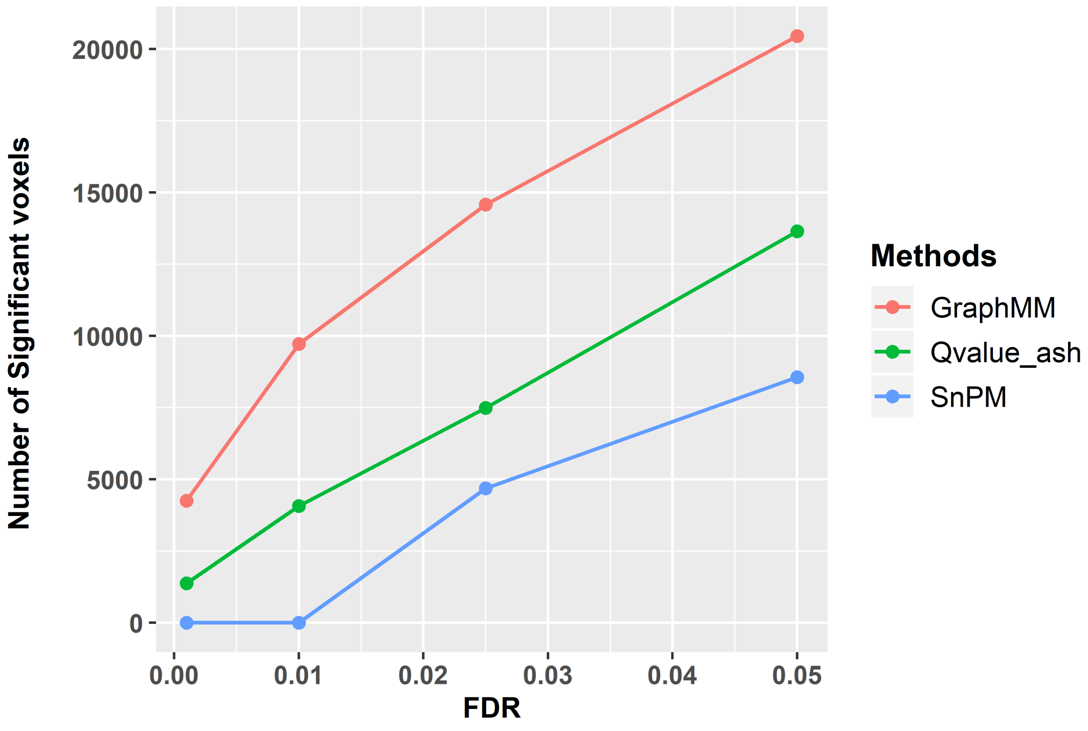

Supplementary Figure S6: Plot of Controlled FDR vs. Number of significant voxels on the whole brain data. The figure confirms the high yield of GraphMM.

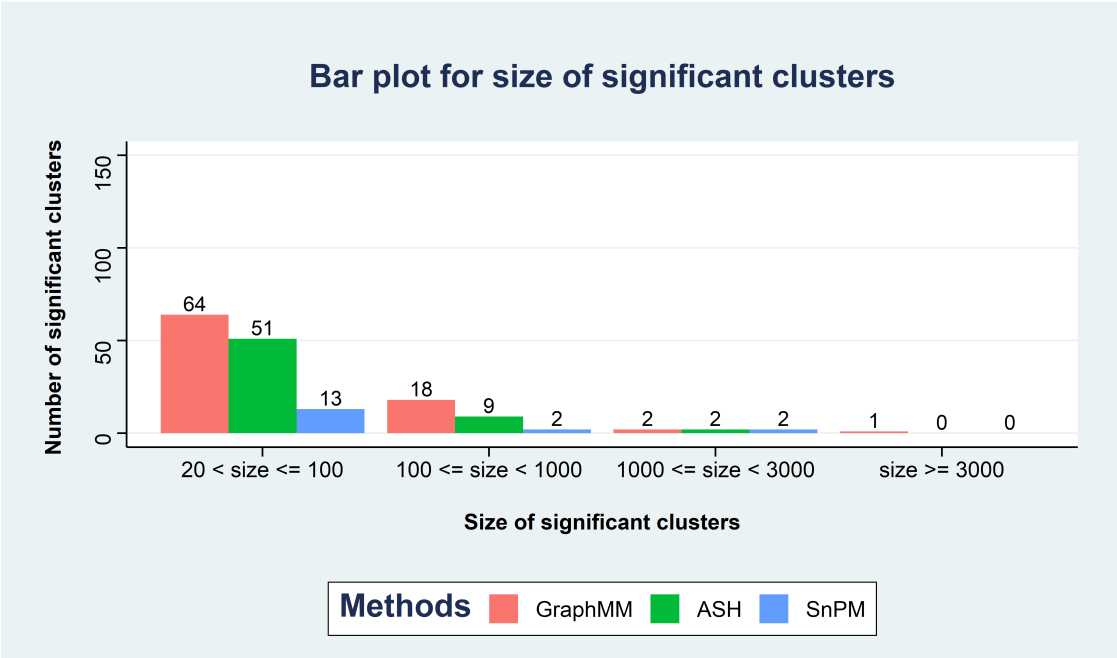

Supplementary Figure S7: Bar plot for summary on the size of significant clusters. By definition, a significant region is a collection of significant voxels that is spatially connected.

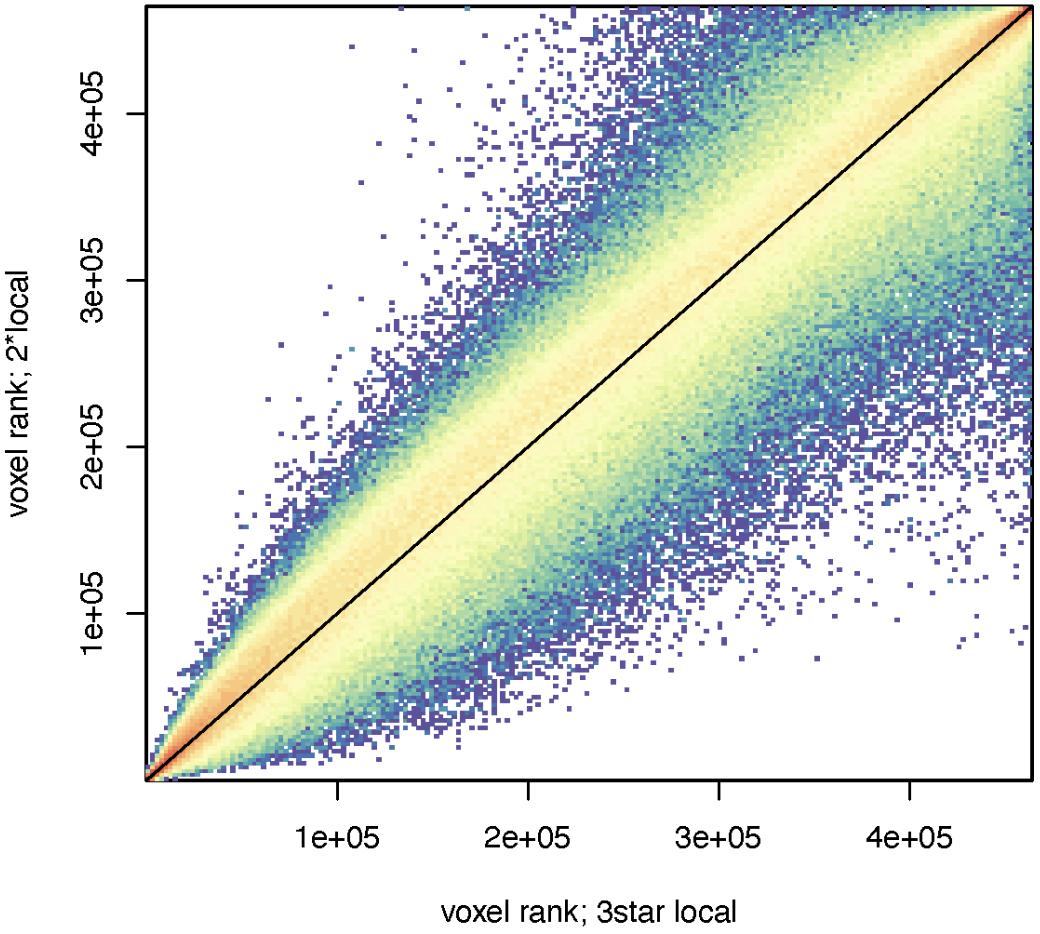

Supplementary Figure S8: Histogram showing the full-brain voxel ranks by GraphMM local FDR when the local graph is a 3d, 7 node star graph (horizontal) or when the local graph is a 2d, within-slice 5 node star graph (vertical). Voxel prioritization is different by the two methods, but there is a great deal of consistency. (Ranks from the bottom, so smallest local FDR (and greatest group difference) is on the bottom left.)

Supplementary Table S4: Brain regions with significant (5% FDR) change in gray matter volume found by GraphMM.

No.	Brain region	# Voxels		Neurological function
		GraphMM	SnPM	
1	Hippocampus	1411	1646	Receives and consolidates new memory about experienced events, allowing for establishment of long-term memories. [?]
2	Parahippocampal Gyrus	1008	410	Involved in episodic memory and visuospatial processing [?].
3	Amygdala	710	365	Plays an essential role in the processing and memorizing of emotional reactions [?]
4	Temporal Gyrus (superior, middle and inferior)	2031	287	Involved in various cognitive processes, including language and semantic memory processing (middle) as well as visual perception (inferior) and sound processing (superior) [?, ?]
5	Putamen	793	12	Linked to various types of motor behaviors, including motor planning, learning, and execution.[?].
6	Fusiform Gyrus	735	308	Influence various neurological phenomena including face perception, object recognition, and reading [?].
7	Temporal Pole (superior, middle)	882	74	Involved with multimodal analysis, especially in social and emotional processing. [?].
8	Precentral Gyrus	829	0	Consists of primary motor area, controlling body's movements. [?].
9	Middle Frontal Gyrus	635	0	Plays essential role in attentional reorienting. [?].
10	Inferior Frontal Gyrus Opercular	573	0	Linked to language processing and speech production. [?].
11	Calcarine	381	22	Where the primary visual cortex is concentrated, processes visual information. [?].
12	Caudate	437	46	Plays essential roles in motor processes and a variety of executive, goal-directed behaviours [?].
13	Insular	275	0	Involved in consciousness, emotion and the regulation of the body's homeostasis [?].
14	Anterior Cingulate	260	0	Plays a major role in mediating cognitive influences on emotion. [?].
15	Supramarginal Gyrus	225	0	Linked to phonological processing and emotional responses. [?].

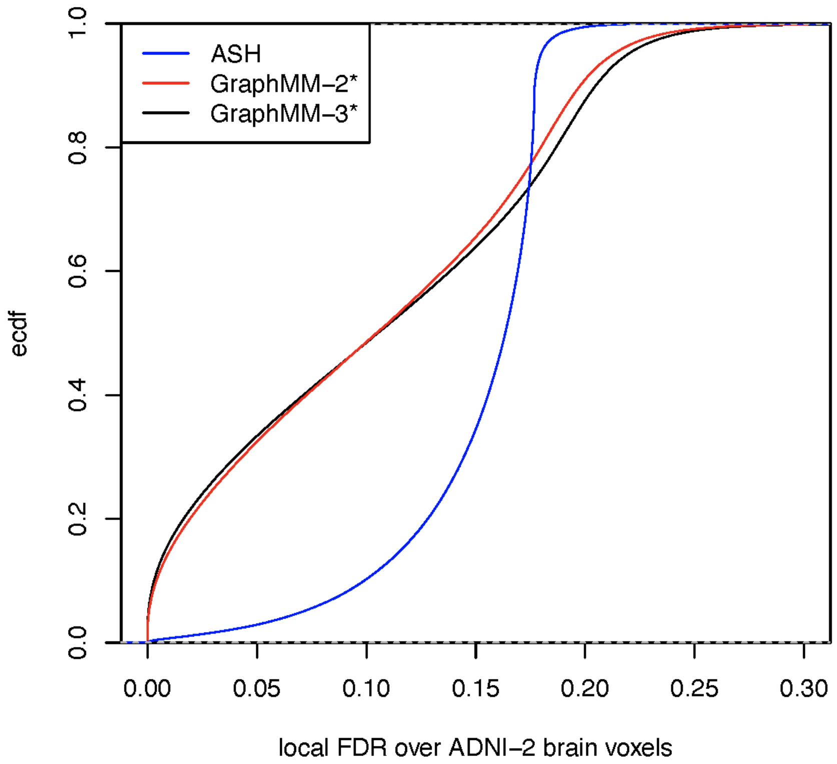

Supplementary Figure S9: The empirical distribution function (ecdf) of voxel-specific local FDR statistics from three computations and over 464258 voxels in the ADNI-2 data (those voxels from the giant component of the filtered lattice graph). The local subgraph in GraphMM is either the one holding all first neighbors (GraphMM-3*) or the one holding all such neighbors in a 2-D coronal slice (GraphMM-2*). Note the black curve rises slightly faster from the origin, indicating more local FDR statistics close to zero for GraphMM-3*. The mean values of the distributions are similar (approximating π_0). ASH statistics, which do not attempt to use the brain structure, are included for comparison.

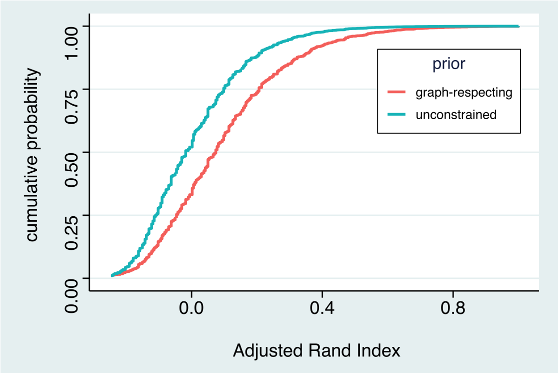

Supplementary Figure S10: Shown are predictive averages of posterior similarity distributions between the generative mean partition and the posterior distribution over partitions for two analysts. For each similarity value s (Adjusted Rand Index), each curve records the predictive average $E\left[P(S(\Psi,\Psi^*) \leqslant s|D)|\mathrm{OK}\right]$, where OK is the event that the true partition Ψ^* is graph-respecting. One analyst uses a prior that ignores the graph; the other uses a graph-respecting prior. The analyst who has regularized posterior computations tends to place more posterior probability near the generative partition.

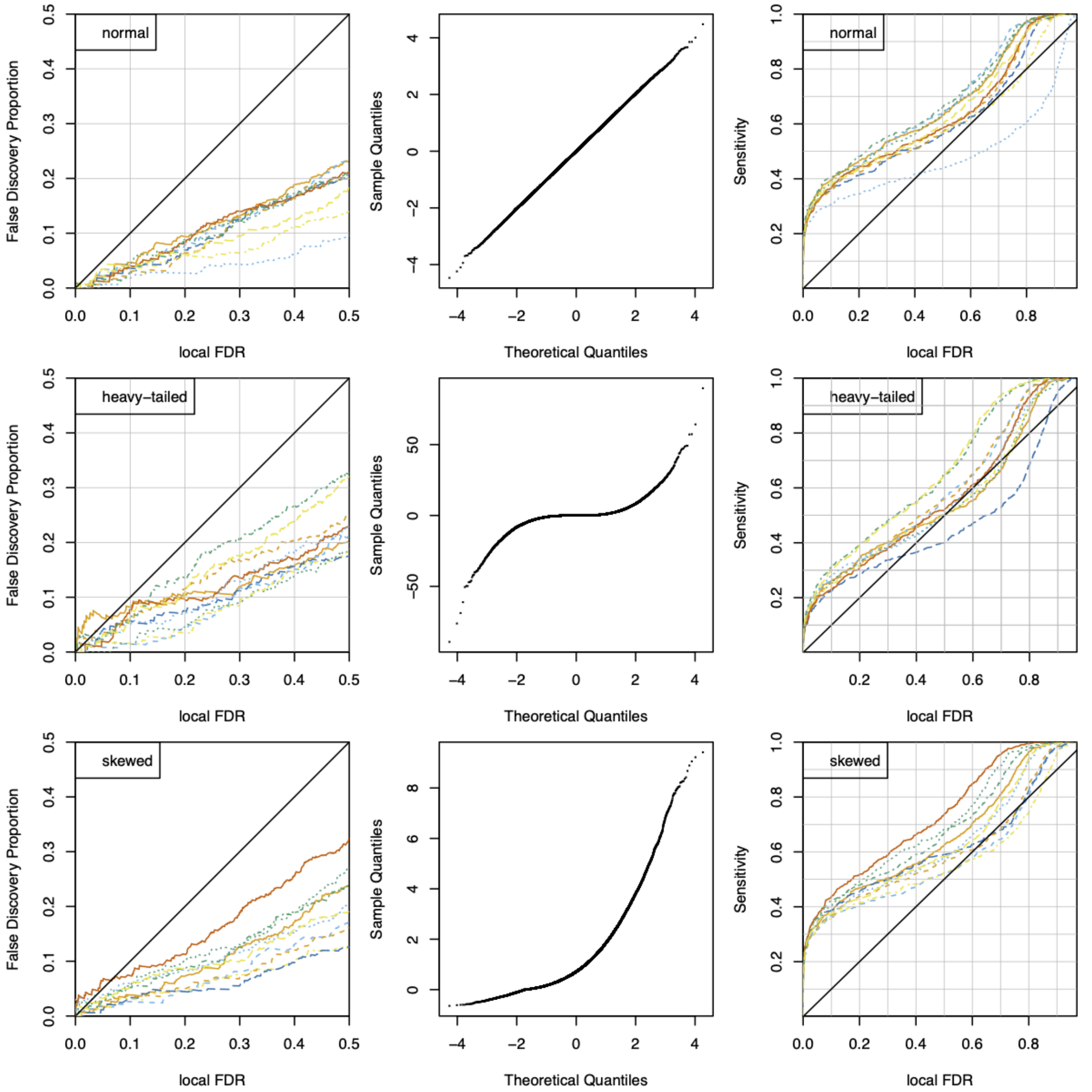

Supplementary Figure S11: FDR control (left), sensitivity (right), and non-normality (center) for three simulation scenarios on a line graph with 1000 nodes. Ten data sets are simulated in each (separate lines); the normal qq plot of data from one of two synthetic groups is shown in the center. GraphMM computed local FDR is on the horizontal on left and right columns.

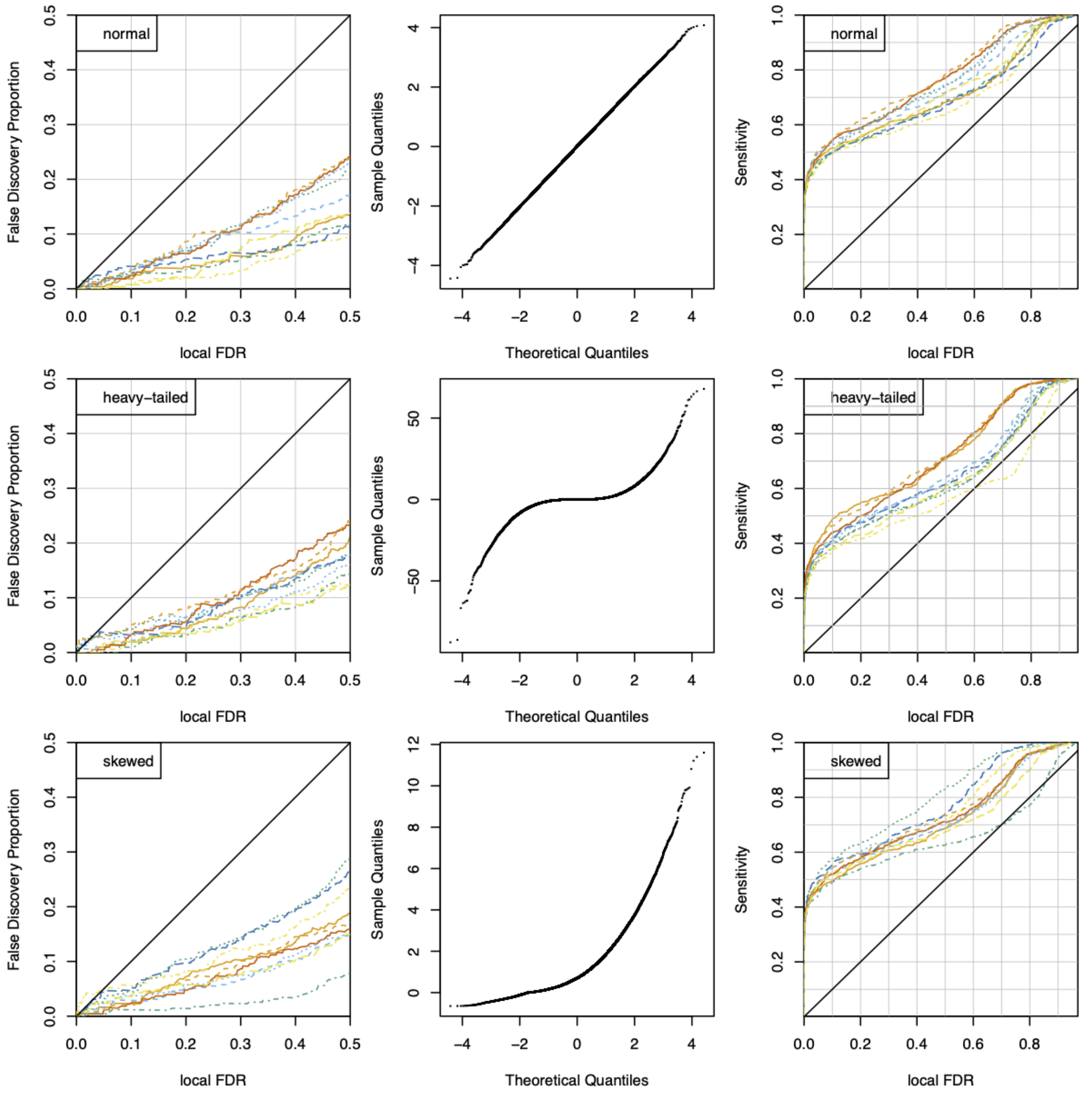

Supplementary Figure S12: Same as Supplementary Figure S11, but with double the sample size per simulated data set, and also some improved FDR control and sensitivity. Results from 10 simulated data sets are shown in each panel (separate lines).
